# Human NLRC4 expression promotes cancer survival and associates with type I interferon signaling and immune infiltration

**DOI:** 10.1172/JCI166085

**Published:** 2024-04-23

**Authors:** Charlotte Domblides, Steven Crampton, Hong Liu, Juliet M. Bartleson, Annie Nguyen, Claudia Champagne, Emily E. Landy, Lindsey Spiker, Christopher Proffitt, Sunil Bhattarai, Anissa P. Grawe, Matias Fuentealba Valenzuela, Lydia Lartigue, Isabelle Mahouche, Jeremy Dupaul-Chicoine, Kazuho Nishimura, Félix Lefort, Marie Decraecker, Valérie Velasco, Sonia Netzer, Vincent Pitard, Christian Roy, Isabelle Soubeyran, Victor Racine, Patrick Blanco, Julie Déchanet-Merville, Maya Saleh, Scott W. Canna, David Furman, Benjamin Faustin

**Affiliations:** 1 University of Bordeaux, Bordeaux, France.; 2 ImmunoConcEpt, CNRS UMR 5164, INSERM ERL 1303, Bordeaux University, Bordeaux, France.; 3 Department of Medical Oncology, University Hospital of Bordeaux, Bordeaux, France.; 4 Discovery Immunology, Johnson & Johnson Innovative Medicine, San Diego, California, USA.; 5 GI and Immune-Oncology DDUs, Takeda Pharmaceuticals, San Diego, California, and Cambridge, Massachusetts, USA.; 6 Buck Institute for Research on Aging, Novato, California, USA.; 7 Department of Medicine, McGill University, Montreal, Québec, Canada.; 8 Department of Immunology, University of Pittsburgh School of Medicine, Pittsburgh, Pennsylvania, USA.; 9 Department of Genetics, University of Pittsburgh School of Public Health, Pittsburgh, Pennsylvania, USA.; 10 INSERM, U1218 ACTION, F-33000, Bordeaux, France.; 11 Comprehensive Cancer Center, Department of Biopathology, Institut Bergonié, Bordeaux, France.; 12 QuantaCell, Hôpital Saint Eloi, IRMB, Montpellier, France.; 13 INRS Santé Biotechnologie, Laval, Québec, Canada.; 14 Pediatric Rheumatology, The Children’s Hospital of Philadelphia and University of Pennsylvania Perelman School of Medicine, Philadelphia, Pennsylvania, USA.; 15 Stanford 1000 Immunomes Project, Stanford School of Medicine, Stanford, California, USA.

**Keywords:** Immunology, Oncology, Cancer immunotherapy, Cellular immune response, Innate immunity

## Abstract

The immune system can control cancer progression. However, even though some innate immune sensors of cellular stress are expressed intrinsically in epithelial cells, their potential role in cancer aggressiveness and subsequent overall survival in humans is mainly unknown. Here, we show that nucleotide-binding oligomerization domain–like receptor (NLR) family CARD domain–containing 4 (NLRC4) is downregulated in epithelial tumor cells of patients with colorectal cancer (CRC) by using spatial tissue imaging. Strikingly, only the loss of tumor NLRC4, but not stromal NLRC4, was associated with poor immune infiltration (mainly DCs and CD4^+^ and CD8^+^ T cells) and accurately predicted progression to metastatic stage IV and decrease in overall survival. By combining multiomics approaches, we show that restoring NLRC4 expression in human CRC cells triggered a broad inflammasome-independent immune reprogramming consisting of type I interferon (IFN) signaling genes and the release of chemokines and myeloid growth factors involved in the tumor infiltration and activation of DCs and T cells. Consistently, such reprogramming in cancer cells was sufficient to directly induce maturation of human DCs toward a Th1 antitumor immune response through IL-12 production in vitro. In multiple human carcinomas (colorectal, lung, and skin), we confirmed that *NLRC4* expression in patient tumors was strongly associated with type I IFN genes, immune infiltrates, and high microsatellite instability. Thus, we shed light on the epithelial innate immune sensor NLRC4 as a therapeutic target to promote an efficient antitumor immune response against the aggressiveness of various carcinomas.

## Abstract 2

Epithelial human NLRC4 promotes patient survival and is associated to Type-I Interferon signaling and immune infiltration in cancer.

## Introduction

Higher vertebrates have developed innate protective mechanisms that can detect stress-induced cues triggered by infection, injury, or carcinogenesis. One example of cytosolic sensors of cellular perturbations are the nucleotide-binding domain and leucine-rich repeat receptors (NLR) family of proteins. Some NLRs can be expressed in both epithelial and immune cells. After sensing cellular cues, NLRs assemble into a molecular scaffold that matures inflammatory caspases (including caspase-1) by a close-proximity mechanism. Such multiprotein complexes are called inflammasomes and, depending on the specific NLR engaged in the machinery, distinct inflammasomes can form in response to a plethora of cellular insults (1). Active caspase-1 can then mediate numerous downstream immune signaling events, including the maturation of inflammatory cytokines (IL-1β and IL-18), pyroptotic cell death, production of inflammatory lipids, and modulation of cellular metabolism (1). NLR family CARD domain–containing 4 (NLRC4) is a crucial component of bacterial innate immune sensing by triggering diverse mechanisms involved in intestinal epithelial cell homeostasis and innate immune responses through production of IL-18, among others, after inflammasome formation (2). In addition to this role in pathogen sensing, NLRC4’s function has recently expanded to include triggering inflammation in sterile conditions through inflammasome assembly after the detection of metabolic dysregulation during aging and the presence of host short interspersed nuclear element (SINE) RNAs (3, 4).

Strikingly, in humans, constitutive NLRC4 hyperactivation through mendelian inheritance of various de novo gain-of-function (GOF) mutations results in a broad array of clinical features, including severe enterocolitis and gut inflammation (5–7), leading to autoinflammatory syndromes collectively termed NLRC4 inflammasomopathies (8). A role for NLRC4 in the pathogenesis of cancer has also been proposed, albeit limited to studies relying on bulk tumor gene expression analysis and mouse models (9). Therefore, its relevance in human cancer and prognosis is scarce, including the critical cell types involved and mechanism of protection. In multiple colorectal cancer (CRC) patient cohorts, we show that loss of NLRC4 protein expression specifically in the tumor, but not the stroma, is associated with aggressive metastatic progression, decreased patient survival, and lower DC and T cell immune infiltrates. At the molecular level, we identified inflammasome-independent functions of NLRC4 in triggering type I IFN signaling and chemokine production in human colon cancer cells to directly induce maturation of DCs toward Th1 polarization in vitro. Hence, expression of human epithelial NLRC4 contributes to mounting an efficient antitumor immune response and protects against aggressive metastatic CRC and potentially others, including lung and melanoma cancers.

## Results

### Loss of gut epithelial NLRC4 expression predicts poor clinical survival in CRC and progression to stage IV metastasis.

To monitor protein expression levels in patient tumor cells and stroma, we utilized an immunofluorescence-based imaging approach using high-throughput analysis of tissue microarrays (TMAs) with samples from healthy controls and patients diagnosed with various clinical stages of CRC. Samples were either collected from the Bergonié Cancer Institute or commercially sourced as a validation cohort (104 and 216 patients, respectively; clinical features of cohorts described in Supplemental Table 1 and Supplemental Figure 2; supplemental material available online with this article; https://doi.org/10.1172/JCI166085DS1; see Methods). The image-based epithelial segmentation of patient tissues used to determine protein levels of NLRC4, IL-1β, and IL-18 expressed in epithelial cells versus stromal cells is described in Supplemental Figure 1A and Methods. Supplemental Figure 1B shows a robust loss of NLRC4 protein expression as well as that of inflammasome-dependent cytokines IL-1β and IL-18 in tumor cells compared with normal tissue (104 patients, Bergonié cohort). Loss of NLRC4 was validated in an independent cohort of CRC patients (*n* = 208, Supplemental Figure 2). This result is consistent with downregulation of *NLRC4* gene expression obtained from The Cancer Genome Atlas (TCGA) transcriptomics analysis of bulk tumors of patients with gastrointestinal cancer, including colon adenocarcinoma (COAD), cholangiocarcinoma (CHOL), and liver hepatocellular carcinoma (LIHC) (Supplemental Figure 1C). Importantly, a strong downregulation of *NLRC4* gene expression was also observed in patient tumors with lung cancers (lung adenocarcinoma [LUAD] or squamous cell carcinoma [LUSC]) (Supplemental Figure 1C); hence, despite great variability among cancer types, the decrease in *NLRC4* in tumors seems to be a generalizable phenomenon. This analysis was extended to other epithelial cell–expressed NLR inflammasomes, and slightly different profiles were observed for *NLRP3*, *NLRP1*, and *NLRP6*, with the most striking being for *AIM2*, the downregulation of which was only observed in LIHC (Supplemental Figures 3 and 4).

**Figure 1:**
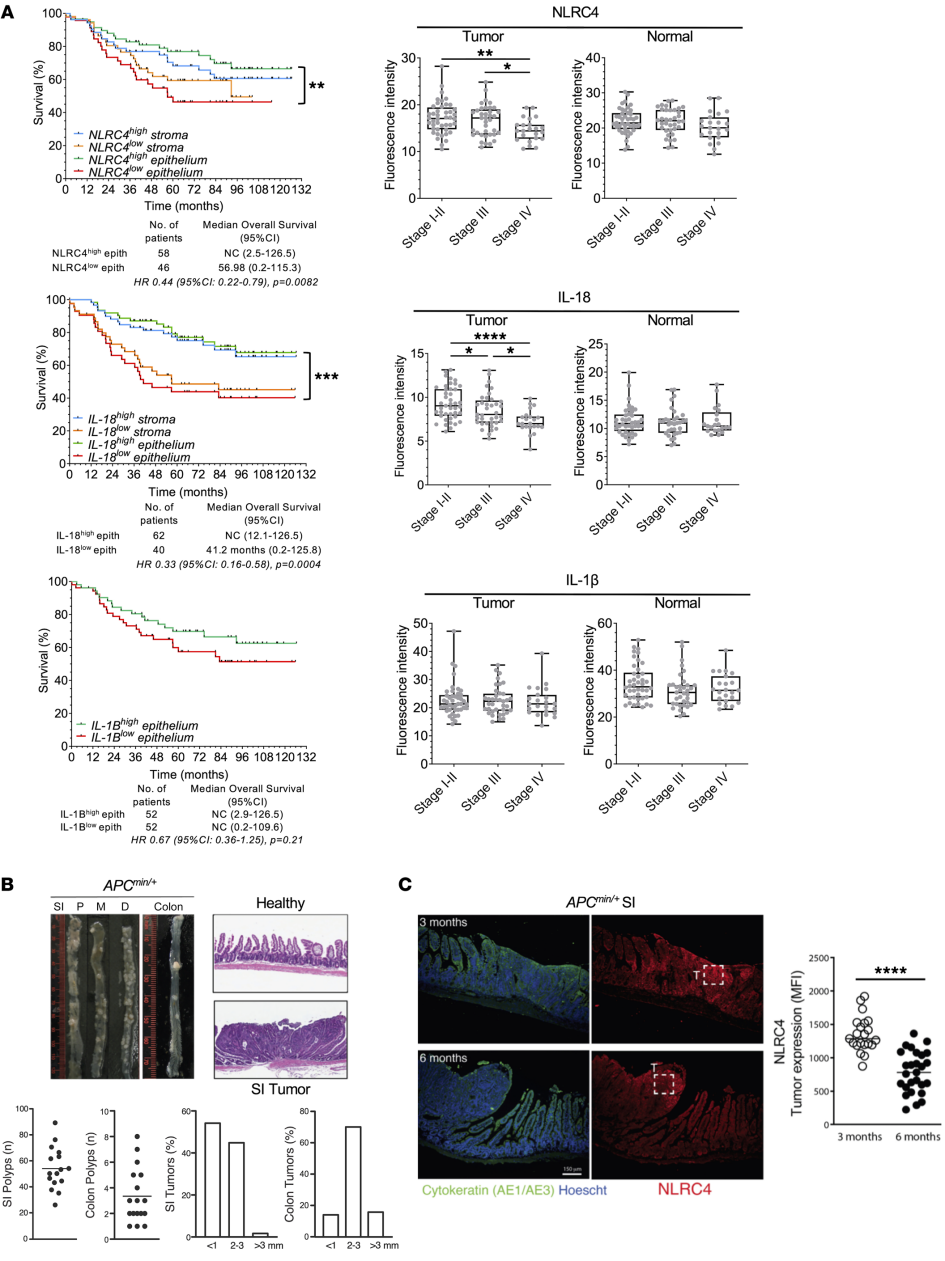
Loss of tumor epithelial NLRC4 is associated with aggressive metastatic stage IV, and decreased overall survival of CRC patients. (**A**) Left: Association between protein expression levels of tumor epithelial NLRC4, IL-18, or IL-1β and patient overall survival in the Bergonié Cancer Institute cohort. When available, patients were stratified based on protein expression levels of NLRC4, IL-1β, or IL-18, as high versus low expression in the colon epithelium (inside the cytokeratin mask) or in the stroma (outside the cytokeratin mask). NC, could not be calculated. A log-rank test stratified according to protein expression was used. Asterisks indicate *P* values between high versus low expression of markers either inside or outside the mask. Right: Protein expression of tumor epithelial NLRC4, IL-18, or IL-1β, in various CRC tumor stages, classified as stage I–II (localized), III (locally advanced), or IV (metastatic disease) (from the Bergonié cohort). COAD, colon adenocarcinoma; READ, rectum adenocarcinoma; CHOL, cholangiocarcinoma; LUAD, lung adenocarcinoma; LUSC, lung squamous cell carcinoma. *P < 0.05; **P < 0.01; ***P < 0.001; ****P < 0.0001 by 1-way ANOVA (parametric) or Kruskal-Wallis (nonparametric) test. (**B**) Top left: Representative image of polyps of a 6-month-old *Apc^Min/+^* mouse in the small intestine (SI) according to their position (P, proximal; M, middle; or D, distal) and in the colon. Top right: H&E staining of healthy SI and polyp-containing section of a 6-month-old *Apc^Min/+^* mouse. Original magnification, ×4. Bottom: Quantification of the number of polyps in the SI (*n* = 17) and the colon of *Apc^Min/+^* mice (*n* = 17), and the size distribution of polyps from 6-month-old *Apc^Min/+^* mice in the SI and colon. (**C**) Representative immunofluorescence images of SI tissue sections at 3 or 6 months of *Apc^Min/+^* mice stained with anti-NLRC4 antibody (red). Anti-cytokeratin (green) and Hoechst (blue) were used to stain epithelial cells and nuclear morphology, respectively. Boxes indicate regions (150 × 150 μm) used to quantify NLRC4 staining in tumor (T) portion of the tissue. Graph shows NLRC4 expression level (MFI) in tumor region of SI at 3 or 6 months in *Apc^Min/+^* mice. Data are mean ± SEM of 21–27 distinct normal/tumor regions, 3 regions per mouse, 7–9 mice per time point. ****P < 0.0001 by 2-tailed Student’s *t* test.

To address the clinical consequences of NLRC4 expression in tumor tissues, the patients’ cohort was stratified based on protein expression levels of NLRC4, IL-1β, or IL-18, as high versus low expression for each marker (above/below median; see Supplemental Figure 5 and Methods), either in the colon epithelium (within the cytokeratin mask) or in the stroma (outside the cytokeratin mask) (see Methods). Patients with high levels of NLRC4 protein expression within the tumor had a better survival compared with those with low expression (HR: 0.44, 95%CI: 0.22–0.79, *P* = 0.0082) (Figure 1A). The median overall survival was 56.98 months for NLRC4^lo^ patients (*n* = 46), while the median was not reached for the NLRC4^hi^ individuals (*n* = 58) within the study period. Variation of stromal NLRC4 protein expression was not associated with a difference in overall survival (HR: 0.79, 95%CI: 0.42–1.46, *P* = 0.45). In contrast with NLRC4, loss of IL-18 protein expression within both epithelial and stromal compartments was associated with a worse outcome (*P* = 0.0004). Interestingly, unlike NLRC4 and IL-18, variation in IL-1β protein expression in the cytokeratin tumor mask was not associated with a difference in survival. Analysis of the independent ClinicalOutcome public data set demonstrated improved survival in NLRC4^hi^ patients using tumor bulk analysis at the gene expression level from patients with CRC (*P* = 0.0169) (Supplemental Figure 6A), as well as in lung cancers (*P* = 0.0002) (Supplemental Figure 6B). Improved survival of NLRC4^normal^ patients was also observed for glioblastoma (*P* = 0.0001), while other cancer types were not significant or missing data (Supplemental Figure 6). Furthermore, consistent with the poor overall survival in NLRC4^lo^ CRC tumors, compared with early clinical stages I–II (*n* = 44) and locally advanced stage III cancer (*n* = 37), a gradual loss of tumor NLRC4 protein expression was further observed in metastatic stage IV cancer (*n* = 23) (stage I–II vs. IV, *P* = 0.0082) in our Bergonié cohort (Figure 1A). This was also the case for IL-18 (stage I–II vs. IV, *P* < 0.0001), but not for IL-1β (Figure 1A). This loss of NLRC4 protein expression in aggressive stage IV was further confirmed in the validation cohort (stage I–II, *n* = 150; stage III, *n* = 47; stage IV = 11) (Supplemental Figure 2). This loss of NLRC4 protein expression across tumor stages is consistent with a significant decrease at the gene expression level for COAD as well as lung cancers (LUAD and LUSC) (bulk tumor analysis from TCGA patient data set; Supplemental Figure 7, A and B). Therefore, gradual loss of NLRC4 protein expression in tumors, but not stromal cells, is associated with poor clinical survival of CRC patients, consistent with the aggressive progression to the metastatic stage. This observation might extend to human lung cancers as well.

To investigate this mechanism in mice, we used a genetically driven model of intestinal early polyp formation spontaneously occurring in *Apc^Min/+^* mice (see Methods). In this model, high numbers of small intestine polyps were observed in *Apc^Min/+^* mice, along with the loss of overall mucosal tissue architecture compared with healthy tissue (Figure 1B). Interrogation of mouse NLRC4 protein expression within the tumor (cytokeratin mask) showed a progressive loss of expression as the tumor grew between 3 and 6 months (Figure 1C). Consistent with this, it was previously shown that absence of NLRC4 expression in *Nlrc4*^–/–^ mice promotes colon tumorigenesis in the azoxymethane (AOM)/dextran sodium sulfate (DSS)–induced inflammatory CRC model wherein tumors appeared aggressive, with invasion of tumor cells below the muscular mucosae (10). Therefore, the gradual loss of NLRC4 expression in the tumor is associated with cancer progression and seems to be conserved between human and mouse.

### Loss of tumor NLRC4 expression is associated with impaired T cell and DC immune infiltrates in cancer patients.

**Figure 2:**
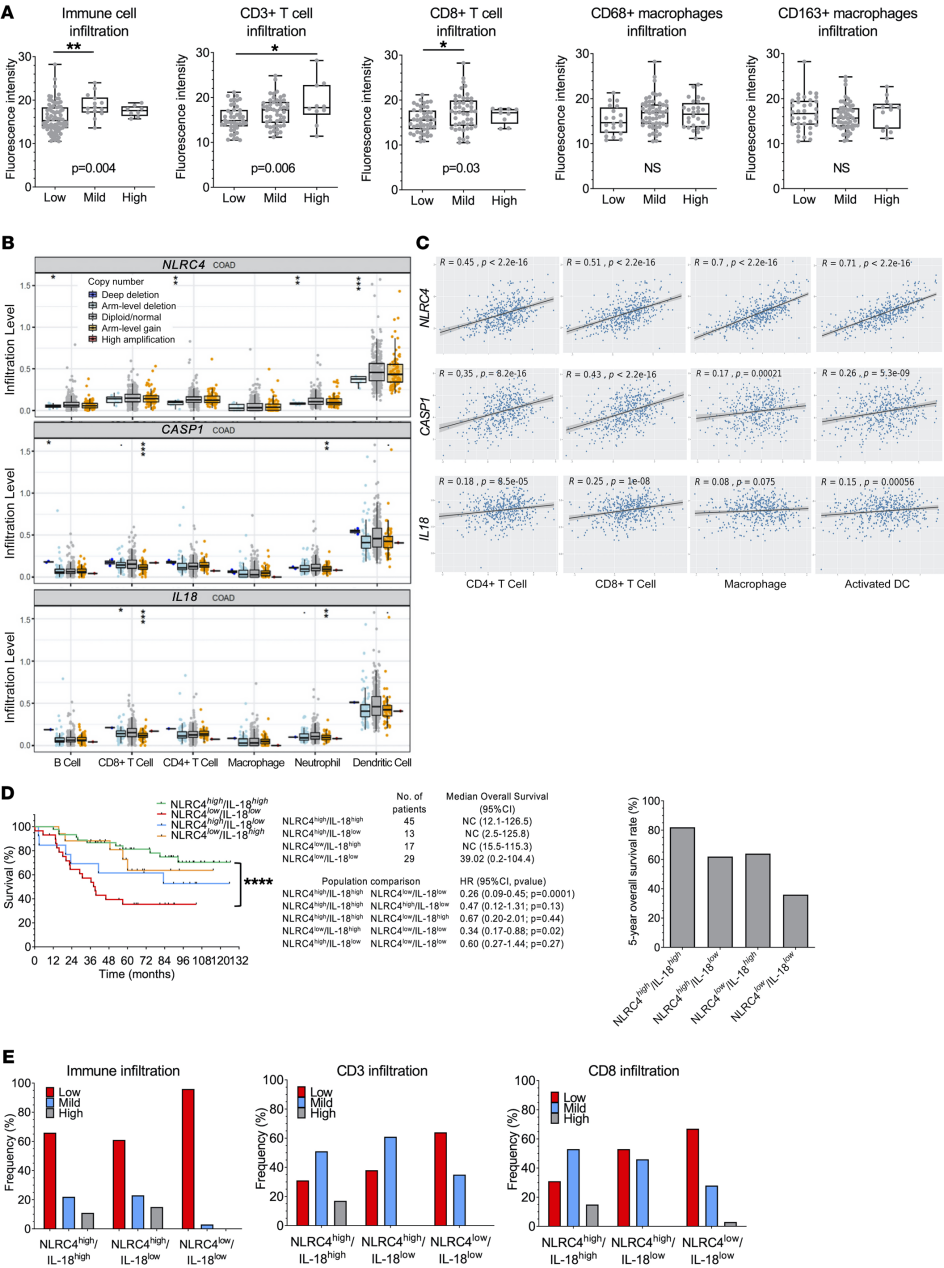
Loss of tumor epithelial NLRC4 protein is associated with low tumor immune infiltration of CD3^+^ T cells and activated DCs. (**A**) Protein expression of tumor epithelial NLRC4 in various clinically defined levels of tumor total immune, total T cell, cytotoxic T cell, CD68^+^ macrophage, or CD163^+^ macrophage infiltration; as determined by immunohistochemistry as low, medium, and high levels of infiltration in tumors (from the Bergonié Cancer Institute cohort). One-way ANOVA (parametric) or a Kruskal-Wallis test (nonparametric) was used to evaluate the correlation between expression intensity and levels of immune infiltrates. (**B**) Associations between *NLRC4*, *CASP1*, or *IL18* somatic copy number alterations and composition of the tumor immune infiltrate, obtained from TCGA cohort and analyzed using TIMER. Box-and-whisker plots are presented to show the distributions of each immune subset at each copy number status in COAD cancer patients. The infiltration level for each category was compared with the normal using 2-sided Wilcoxon’s rank-sum test. (**C**) Correlation coefficient and *P* values between *NLRC4*, *CASP1*, or *IL18* transcripts and levels of immune infiltration for various immune cell subsets in COAD patients, obtained from TCGA cohort and analyzed using TIMER. (**D**) Association between protein expression levels of tumor epithelial NLRC4 and IL-18 with patient overall survival in the Bergonié cohort. Patients were stratified based on protein expression levels of NLRC4 and IL-18 (left) as high versus low expression in the colon epithelium (inside the cytokeratin mask). NC, could not be calculated. A log-rank test stratified according to protein expression was used. Right: 5-year overall survival rate (%). (**E**) Frequency of patients with low, mild, or high tumor immune infiltrates (as pathologically characterized by immune infiltration, CD3^+^ T cells, or CD8^+^ T cells) among 3 different population of patients expressing high or low levels of tumor epithelial NLRC4 and/or IL-18 (from the Bergonié cohort). COAD, colon adenocarcinoma. *P < 0.05; **P < 0.01; ***P < 0.001; ****P < 0.0001.

Based on its central role in the gut mucosal innate immune response, we hypothesized that NLRC4 may regulate the antitumor immune response in patients with CRC. To test this, we interrogated immune infiltrates within tumors of our TMA cohort. We observed that compared with patients with high immune infiltrate (*n* = 8), patients with low immune infiltrate (*n* = 82) had concomitant lower expression of tumor NLRC4 (*P* = 0.004). This was more pronounced for total T cells (CD3^+^, *P* = 0.006) than in CD8^+^ T cells (*P* = 0.03) and was not significant for CD68^+^ and CD163^+^ macrophages (Figure 2A). To validate and extend this observation, we performed a TCGA cohort analysis to determine the abundance of additional tumor-infiltrating immune cell populations in COAD (9) (Figure 2B). Consistent with the results obtained from our TMAs, patients harboring *NLRC4*-null tumors (through genetic arm-level deletion) exhibited decreased CD4^+^ T cell tumor infiltration versus diploid normal tumors (*P* < 0.01). This was also true for neutrophils (*P* < 0.01) and even more significant for DCs (*P* < 0.001). Interestingly, this association of *NLRC4* with impaired infiltration of CD4^+^ T cells and DCs was not observed for *CASP1* (caspase-1) nor *IL18* inflammasome genes (Figure 2B), and only for *IL1B* with DCs (*P* < 0.01) (Supplemental Figure 8). In addition, the level of *NLRC4* gene expression correlated strongly with immune infiltration of activated DCs (partial correlation *r* = 0.71, *P* = 2.2 × 10^–16^), and CD4^+^ and CD8^+^ T cells to a lesser extent (Figure 2C). To determine the prognostic relationship of both tumor NLRC4 and IL-18 protein expression combined, our TMAs of CRC patients were stratified according to the protein expression levels of NLRC4 and IL-18 as high versus low protein expression in epithelial cancer cells (see Methods). NLRC4^lo^IL-18^lo^ patients had a strong decreased survival compared with NLRC4^hi^IL-18^hi^ individuals (HR: 0.26, 95%CI: 0.09–0.45, *P* = 0.0001) (Figure 2D). Median overall survival was 39.02 months for NLRC4^lo^IL-18^lo^ patients. Interestingly, there was no difference in overall survival between NLRC4^hi^IL-18^hi^ and NLRC4^hi^IL-18^lo^ patients (*P* = 0.13) (Figure 2D). Consistent with these results, the loss of IL-18 with preserved NLRC4 tumor expression (NLRC4^hi^IL-18^lo^) was associated with an intermediate survival in comparison with either NLRC4^hi^IL-18^hi^ or NLRC4^lo^IL-18^lo^ patients (5-year survival rates of 82% for NLRC4^hi^IL-18^hi^, 62% for NLRC4^hi^IL-18^lo^, and 35% for NLRC4^lo^IL-18^lo^) (Figure 2D). These results suggest that part of the prognostic effect of NLRC4 tumor expression might involve IL-18 inflammasome–independent mechanisms. To test this, we investigated the effect of these stratifications on immune and T cell infiltrations in our TMAs (Figure 2E). Consistent with the analysis of overall survival, NLRC4^lo^IL-18^lo^ patients were characterized by a decrease in mild and high immune infiltrates compared with the NLRC4^hi^IL-18^hi^ population, including CD3^+^ and CD8^+^ T cells. Importantly, no difference in immune infiltration was observed between NLRC4^hi^IL-18^hi^ and NLRC4^hi^IL-18^lo^ populations, and the loss of NLRC4 tumor expression within the IL-18^lo^ population reduced mild infiltration to low levels of both CD3^+^ and CD8^+^ T cells in tumors (~20% in frequency, respectively). Therefore, altogether, high tumor NLRC4 expression tracks with immune infiltration, including DCs and T cells, independently of tumor IL-18 variation, and this is consistent with better clinical survival of patients.

### NLRC4 expression in human cancer cells mediates a type I IFN reprogramming, and is associated with high microsatellite instability in patient tumors.

**Figure 3:**
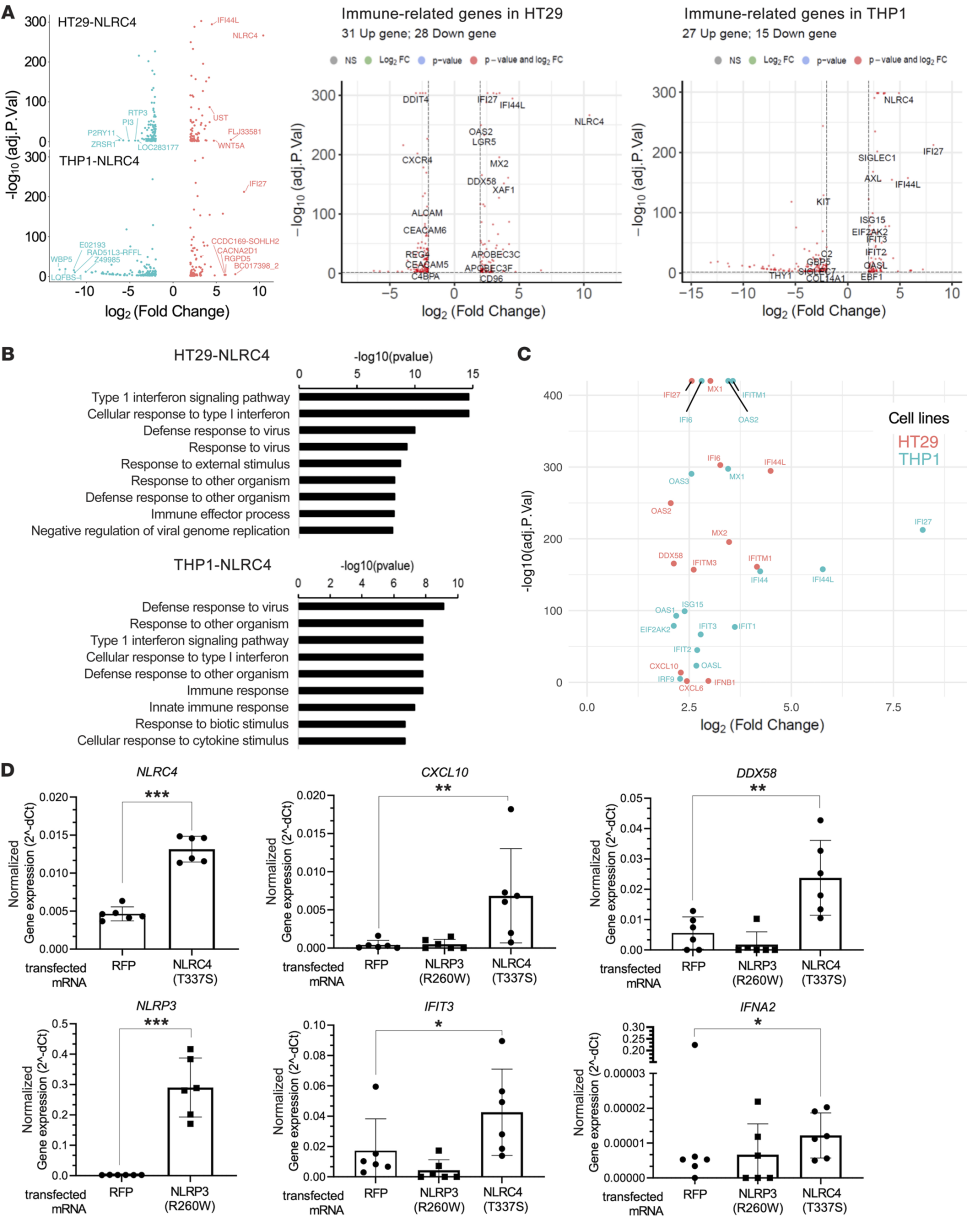
NLRC4 expression in cancer cells triggers an immune transcriptional program. (**A**) Left: Volcano plots of differentially expressed genes in HT29-NLRC4 or THP1-NLRC4 versus mock control cell lines, as measured by RNA-seq analysis. Differential gene expression performed with DeSeq2. The *x* axis shows the log_2_-transformed fold change of NLRC4-overexpressing lines over control, and the *y* axis is the –log_10_ transformation of the adjusted *P* values. The 10 genes most downregulated (blue) or upregulated (red) are included. In the middle, volcano plots of differentially expressed immune-related genes in the HT29-NLRC4 cell line; or to the right, in the THP1-NLRC4 cell line. (**B**) Gene Ontology analysis using KEGG pathway of significant upregulated genes (*P* < 0.05; fold change > 2) in both NLRC4-expressing cell lines. (**C**) Dot plot representing NLRC4-induced type I IFN genes from both cell lines (HT29-NLRC4, red; THP1-NLRC4, blue), with fold changes of gene expression and associated *P* values. (**D**) mRNA transfections of NLRC4 (T337S), or NLRP3 (R260W), or control red fluorescent protein (RFP), in human primary monocytes. Normalized gene expression levels by qPCR of *NLRC4* and *NLRP3* shown as control (left), or type I IFN genes induced by each mRNA transfection as indicated (right). Data are mean of 2 different donors pooled ± SD (*n* = 3 independent mRNA transfections per construct); all transfected constructs were compared to the RFP control using Dunnett’s test. *P < 0.05; **P < 0.01; ***P < 0.001.

To better define the NLRC4 inflammasome–independent mechanisms underlying survival of patients, we stably expressed NLRC4 in colon and monocytic cancer cell lines and conducted whole-genome transcriptomics analysis by RNA-seq (Figure 3A and Supplemental Figure 9, A and B; see Methods). As a control, NLRC4-mCherry expression in the THP1 cell line versus mCherry control did not induce inflammasome activation, as measured by IL-1β cytokine secretion, whereas strong release was triggered by Needle treatment, a specific trigger of the human NLR family of apoptosis inhibitory protein (NAIP)/NLRC4 inflammasome, as expected (Supplemental Figure 9C). The HT29-NLRC4 cell line did not display any changes in cell proliferation or migration compared to mock control (Supplemental Figure 9D). Compared with control, overexpression of NLRC4 in colon HT29 cells induced transcriptional upregulation of 102 genes and downregulation of 127 genes, while 102 genes were upregulated and 144 downregulated in THP1-NLRC4 monocytic cells (based on a fold-change cutoff of 2, *P* < 0.05) (Figure 3A). Among those, approximately one-third were immune-related genes upregulated in both cell lines (Figure 3A). Kyoto Encyclopedia of Genes and Genomes (KEGG) pathway analysis of significant upregulated genes identified “Type-I interferon signaling pathway” as the most significant pathway commonly induced in both cell lines (Figure 3B). Figure 3C illustrates the gene composition of this pathway in both NLRC4-expressing cell lines. Expression of type I IFN genes induced by NLRC4 was further confirmed in human primary monocytes by using mRNA transfections of NLRC4 GOF mutations (previously shown to induce constitutive activation). Both NLRC4 (T337S) and NLRP3 (R260W) neotranscripts were expressed (Figure 3D), leading to upregulation of the top type I IFN genes (*CXCL10*, *DDX58*, *IFIT3*, and *IFNA2*) commonly identified previously in NLRC4 stable cell lines (Figure 3D). Upregulation of these genes was observed in response to the NLRC4 (T337S), but not NLRP3 (R260W), GOF mutation. We also confirmed the upregulation of type I genes *IFNA2* and *IFNB1* using another NLRC4 GOF mutation (V341A), in contrast again with the NLRP3 (R260W) GOF (Supplemental Figure 10), whereas both neotranscripts were expressed and functional to induce caspase-1 activity and IL-18 secretion, as expected (Supplemental Figure 10). Remarkably, the caspase-1 inhibitor YVAD had no effect on the NLRC4 (V341A)–mediated increase in *IFNA2* and *IFNB1* gene expression (Supplemental Figure 10). Consistent with these results obtained with NLRC4 (T337S) mRNA transfections in human primary monocytes, the induction of a broad type I IFN gene signature (encompassing 28 genes validated in patients with IFN-mediated diseases described in ref. 11) was further confirmed in the THP1-NLRC4 (T337) stable cell line versus WT NLRC4 (Supplemental Figure 11A). These type I IFN–stimulated genes were confirmed to be also upregulated in NLRC4 (T337S) patient monocytes versus healthy controls, but not in NLRP3 (G569R) patient monocytes (Supplemental Figure 11B). Unbiased gene set enrichment analysis of RNA-seq revealed a top enrichment of the “interferon alpha response” pathway in THP1-NLRC4 (T337S) cells versus WT NLRC4 (Supplemental Figure 11C). Lastly, treatment of human primary monocytes with Needle (at 0.1 ng/mL) used to activate endogenous NLRC4 was able to recapitulate the upregulation of the top type I IFN genes observed in the NLRC4-expressing cell lines (including *IFI44*, *IFI44L*, *CXCL10*, and *IFIT3*) and after NLRC4 (T337S) mRNA transfection in human primary monocytes (Supplemental Figure 12). Importantly, in this condition we were not able to detect any significant increase in inflammasome activation readouts (caspase-1 activity and IL-18 secretion). As a positive control, robust inflammasome activation could be detected at a higher Needle concentration (1 ng/mL), although the levels of type I IFN genes were not increased compared to the lower Needle concentration (0.1 ng/mL). Altogether, the combination of these various cellular models indicates that NLRC4 expression and activation can trigger a type I IFN transcriptional program, without robust canonical inflammasome activation.

**Figure 4:**
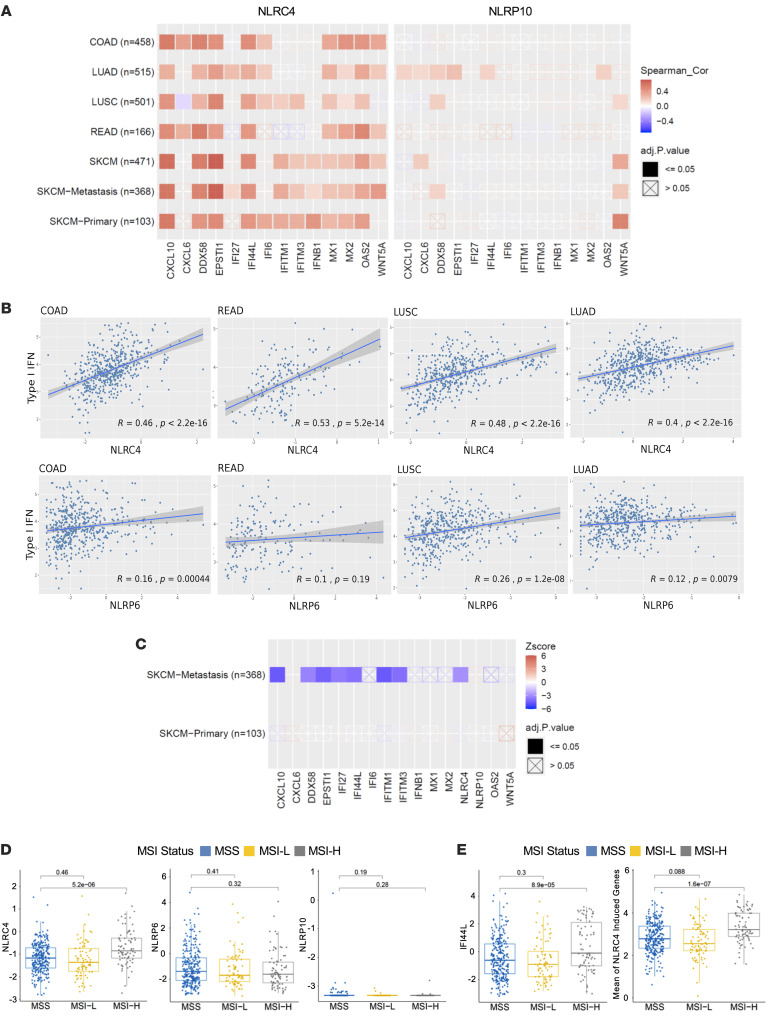
*NLRC4* expression correlates with type I IFN gene signature, MSI^hi^ patient tumors, and is associated with decreased risk of metastasis. (**A**) Correlation analysis between expression of the top type I IFN genes upregulated in the HT29-NLRC4 cell line and *NLRC4* or *NLRP10*, in either CRC patients (COAD, READ), lung cancer patients (LUSC, LUAD), or skin cutaneous melanoma (SKCM, primary and metastasis). (**B**) Correlation analysis between expression of the broader type I IFN gene signature (see Methods) and *NLRC4* or *NLRP6* in CRC patients (COAD, READ), or in lung cancer patients (LUSC, LUAD). Patient data sets from TCGA cohort; Spearman’s correlation coefficient *R* and *P* values are indicated (**A** and **B**). (**C**) Clinical outcome of the top type I IFN genes upregulated in the HT29-NLRC4 cell line (including *NLRC4* or *NLRP10*) in SKCM primary versus metastasis. Patient data sets from TCGA cohort; *z* scores were determined by using TIMER 2.0 and reflect clinical outcome for each gene (blue: decreased risk *P* < 0.05, *z* < 0; red: increased risk *P* < 0.05, *z* > 0), with adjusted *P* values indicated. (**D**) Association between gene expression of NLR family members (*NLRC4*, *NLRP6*, or *NLRP10*) and microsatellite instability (MSI) status. (**E**) Association between type I IFN genes (*IFI44L*, or the gene set encompassing the top 14 NLRC4-induced IFN genes from cell lines) and MSI status in COAD patient tumors. Patient data sets from TCGA cohort, with adjusted *P* values indicated. MSS, MSI stable; MSI-L, MSI low; MSI-H, MSI high; COAD, colon adenocarcinoma; READ, rectum adenocarcinoma; LUAD, lung adenocarcinoma; LUSC, lung squamous cell carcinoma.

To confirm and extend this finding in human disease, we observed that *NLRC4* expression (but not *NLRP10* used as control) strongly correlated with expression of the type I IFN genes induced in the NLRC4-expressing cell lines (as described in Figure 3C) in various patient cancer samples (colon, lung, and melanoma) (Figure 4A). To further validate this, we found that *NLRC4* expression (but not *NLRP6* used as control) was also correlated with a broader unbiased type I IFN gene signature (encompassing 59 genes, see Methods) in colon COAD and rectum adenocarcinoma (READ) patient tumors (*r* = 0.46, *P* < 2.2 × 10^–16^; *r* = 0.53, *P* = 5.2 × 10^–14^, respectively), as well as in LUSC and LUAD patient lung tumors (Figure 4B). We extended the same analysis to other NLR-family members and observed that *NLRC4* expression shows the highest correlation with the type I IFN signature overall across most tumor types (*r* > 0.5), whereas most of the other NLRs show low to no correlation (Supplemental Figure 13). Importantly, consistent with our finding showing a role for NLRC4 in protecting against metastatic progression in CRC, expression of *NLRC4* (but not *NLRP10*) and type I IFN genes was associated with a significantly lower risk of metastasis in patients with skin cutaneous melanoma (SKCM) (Figure 4C). In human CRC, mismatch repair–deficient (MMRD) patient tumors have been shown to have better antitumor immunity with cytotoxic T cell infiltration and response to immune checkpoint blockade (ICB) (12). An enriched immune hub within MMRD tumors has been identified, composed of activated T cells and malignant cells expressing IFN-stimulating genes and CXCR3 ligands (12). Since our results demonstrate that NLRC4 expression in colon cancer cells mediates type IFN signaling and is associated with T cell infiltration and better prognosis in patients, we hypothesized that NLRC4 expression might be associated with microsatellite instability (MSI) in patient tumors. To test this hypothesis, we utilized TCGA data set and observed that *NLRC4* expression was significantly higher in MSI^hi^ versus MSI^lo^ COAD tumors, in contrast with other NLR family members, including *NLRP6* and *NLRP10* (Figure 4D). By extension, we confirmed that *IFI44L* expression (top type I IFN gene upregulated in NLRC4-expressing cell lines) was significantly higher in MSI^hi^ tumors along with the larger gene set encompassing the top 14 type I IFN genes upregulated in NLRC4-expressing cell lines (Figure 4E). Hence, NLRC4 expression can trigger a type I IFN immune reprogramming in human cancer cells that is observed in patient tumors from various cancer types and confers lower risk of metastatic progression.

### NLRC4 expression is associated with DC and CD4^+^ and CD8^+^ T cell immune infiltration in patient tumors.

**Figure 5:**
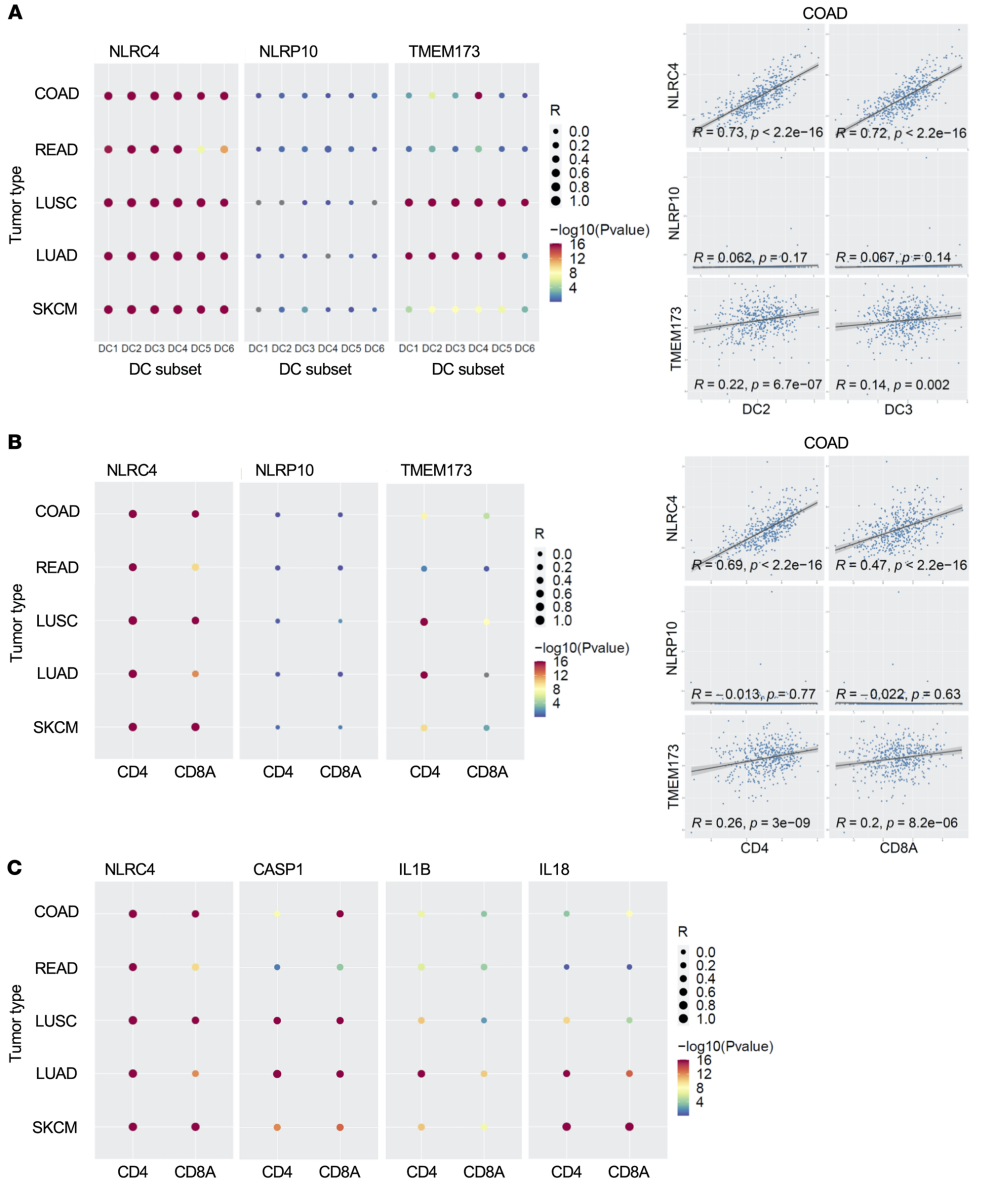
*NLRC4* expression is associated with DC2 and DC3 cells and CD4^+^ and CD8^+^ T cell tumor infiltrates in cancer patients. (**A**) Left: Expression correlation between *NLRC4*, *NLRP10*, or *TMEM173* (STING) and various tumor-infiltrating DC subsets in CRC (COAD, READ), lung cancer (LUSC, LUAD), or melanoma (SKCM). The various DC subset gene signatures used (for DC1–DC6) were obtained from scRNA-seq of human blood. Right: Scatter plots showing correlation of gene expression between *NLRC4*, or *NLRP10*, or *TMEM173*, and DC2 or DC3 gene signatures in COAD patient tumors. COAD data sets used were obtained from TCGA cohort. (**B**) Left: Gene expression correlation between *NLRC4*, *NLRP10*, or *TMEM173* (STING) and *CD4* or *CD8A* in patient tumors; correlation coefficient *R* is represented by the size of dot, and log_10_(*P* value) is represented by the color of the dot. Right: Scatter plots showing correlation of gene expression between *NLRC4*, or *NLRP10*, or *TMEM173* and *CD4* or *CD8A* in COAD patient tumors. Correlation coefficient *R* and *P* values are indicated. (**C**) Gene expression correlation between *NLRC4*, *CASP1*, *IL1B*, or *IL18* and *CD4* or *CD8A* in patient tumors. For **A**–**C**, data analysis was performed using TCGA patient database cohort; correlation coefficient *R* is represented by the size of the dots, and log_10_(*P* value) is represented by the color of the dot. COAD, colon adenocarcinoma; READ, rectum adenocarcinoma; LUAD, lung adenocarcinoma; LUSC, lung squamous cell carcinoma; SKCM, skin cutaneous melanoma.

Type I IFN signaling is critical in priming professional antigen-presenting cells (APCs), including DCs, for tumor antigen presentation and costimulation of T cells. It also provides the cues for Th1 polarization and T cell licensing for tumor cell killing (13). Analysis of the multiple human DC subsets (14) showed the strongest correlations of *NLRC4* expression with DC2, DC3, and DC4 subsets in COAD (*r* = 0.69, 0.71, and 0.64, respectively) as well as in READ patients. Those same 3 subsets were also the most correlated ones in lung cancer (LUSC: *r* = 0.81, 0.78, and 0.81 respectively; similar magnitude for LUAD) as well as in SKCM (Figure 5A). Strikingly, the same analysis performed with *STING/TMEM173* expression (well-characterized type I IFN signaling mediator) provided much lower coefficient correlations than *NLRC4* for those DC2, DC3, and DC4 subsets in both colon and melanoma patient tumors, indicating the role of *NLRC4* expression as a critical driver of DC tumor infiltration in multiple cancer types. Correlation analysis between infiltration of DC2 or DC3 subsets and *NLRC4* expression in COAD patient tumors showed robust associations for either of these cell subsets (*r* = 0.73, *P* < 2.2 × 10^–16^; *r* = 0.72, *P* < 2.2 × 10^–16^ for DC2 and DC3, respectively), in comparison with *NLRP10* and *TMEM173* (Figure 5A). An extended analysis of additional type I IFN inducers showed that *NLRC4* expression had the highest correlation overall across the various DC subsets and tumor types, followed by *IFIH1*, *RIGI*, *TMEM173*, and finally *ADAR* (Supplemental Figure 14). To further evaluate the robustness of these results, we leveraged multiple DC signatures from immune cell deconvolutional tools where signatures were derived from bulk RNA-seq analysis of sorted cells. Overall, we obtained higher correlations of those DC signatures with *NLRC4* compared with *TMEM73* expression in CRC and lung cancers (Supplemental Table 2). Notably, the molecular features of DC2 and DC3 subsets by single-cell RNA-seq (scRNA-seq) analysis have suggested an inflammatory monocyte phenotype and type I IFN signaling/antigen presentation functions (14). Aligned with those molecular features, DC2 and DC3 subsets mediate CD4^+^ and CD8^+^ T cell proliferation (14). Consistent with those observations, *NLRC4* expression (which we found associated with DC2/DC3 subsets in cancer tumors) correlated strongly with *CD4* expression (*r* = 0.69, *P* < 2.2 × 10^–16^) in COAD patient tumors, and *CD8A* to a lesser extent (*r* = 0.47, *P* < 2.2 × 10^–16^) (Figure 5B). Similar results were found for lung and melanoma cancers (Figure 5B). As a control, neither *NLRP10*, *TMEM73* (Figure 5B), nor downstream inflammasome cytokines *IL1B* and *IL18* or *CASP1* (Figure 5C, Supplemental Figures 15 and 16, and Supplemental Table 3) correlated with *CD4* or *CD8A* expression to the same extent as *NLRC4* expression. Therefore, consistent with its association with type I IFN signaling genes observed in our previous analyses, *NLRC4* expression strongly correlates with infiltration of antigen-presenting DC phenotypes and CD4^+^ and CD8^+^ T cells in colon and lung patient tumors.

### NLRC4 expression in human cancer cells mediates the release of type I IFN chemokines and myeloid growth factors to directly induce maturation of DCs in vitro.

**Figure 6:**
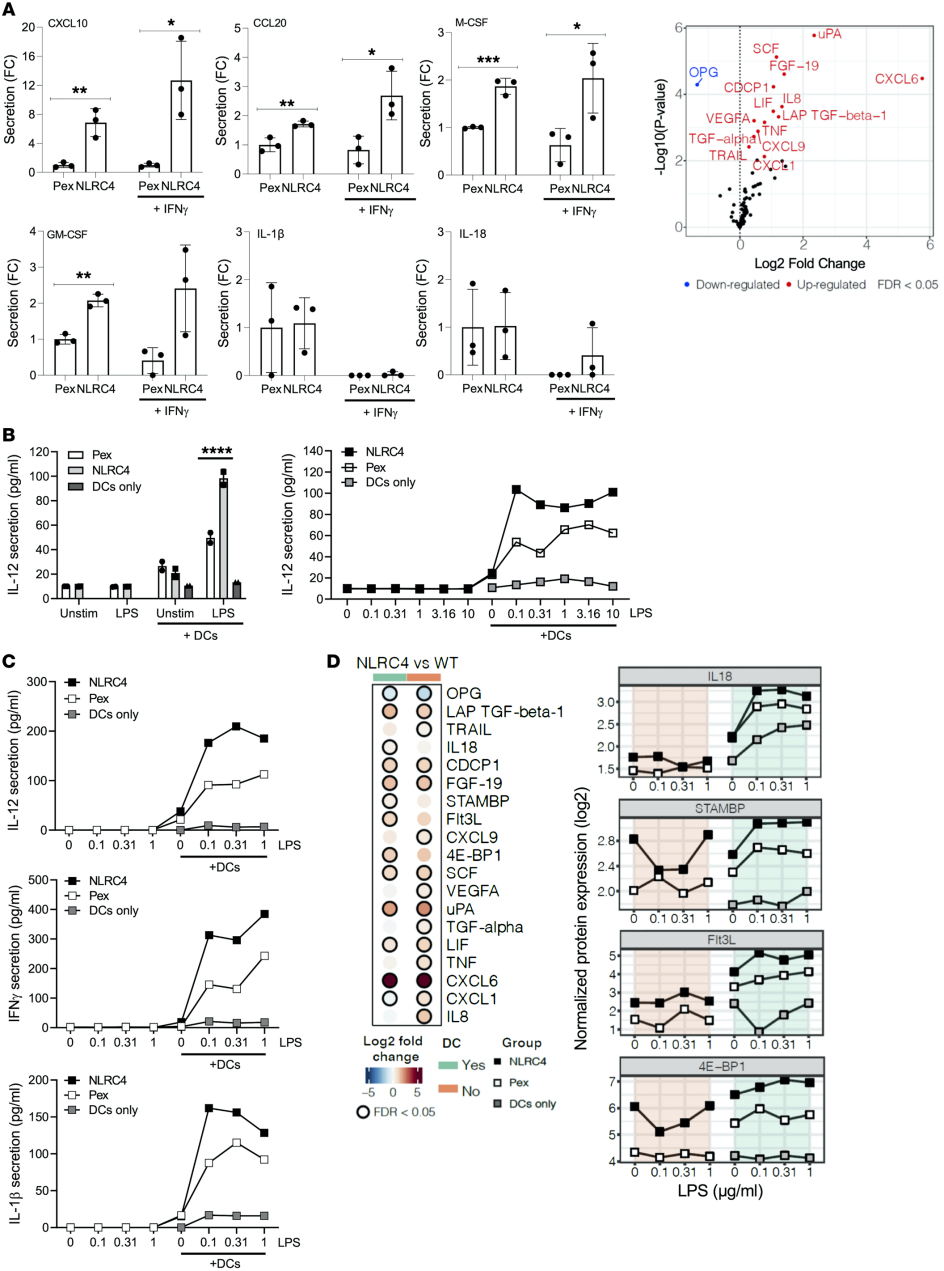
NLRC4 expression in cancer cells mediates the release of type I IFN chemokines and myeloid growth factors to induce maturation of human primary DCs toward a Th1 immune response. (**A**) Left: HT29-NLRC4 cells or HT29-pEx control cells were cultured in Boyden chambers in the presence or not of IFN-γ, and release of the indicated immune mediators (chemokines, myeloid growth factors, cytokines) was measured in the top chamber by ELISA. Right: Volcano plot of differentially secreted proteins by HT29-NLRC4 versus mock control cell lines as measured by Olink Proteomics. *P* values were adjusted by multiple testing using the Benjamini-Hochberg method (see Methods). Data presented as mean ± SD (*n* = 3). (**B**) IL-12 in cell culture supernatants measured by ELISA from cultures of HT29-NLRC4 cell line alone, HT29-pEx control cell line alone, or cocultured with primary DCs isolated from human blood, with or without LPS (0.1 μg/mL [left], or various concentrations in μg/mL [right]). Cocultures of HT29/DCs (1:1.2 ratio) were maintained for 24 hours in the presence or not of LPS. Data presented as mean ± SD (duplicates), representative of 2 donors with similar pattern. (**C**) Same experiment as in **B**, but extended to a broader cytokine array as indicated by using MSD. Data representative of 2 donors with similar patterns. *P < 0.05; **P < 0.01; ***P < 0.001; ****P < 0.0001 by unpaired, 2-tailed Student’s *t* test (**A**) or 2-way ANOVA with Šidák’s post hoc test for NLRC4 vs. pEx (**B**). (**D**) Same experiment as in **B**, but additional differentially secreted proteins as measured by Olink Proteomics. Heatmap in the left panel indicates the log_2_(fold change) between HT29-NLRC4 and WT cells (pEx control), in cocultures with DCs (green) or not (orange), in the presence or not of LPS. Black circles indicate statistically significant changes after multiple testing. Plots to the right show the normalized protein expression values for the various markers, cocultured or not with DCs, with or without LPS.

Based on the results obtained from patient tumors and the fact that HT29-NLRC4 human cancer cells are reprogrammed toward type I IFN signaling, we hypothesized that key immune mediators and growth factors could be released from these cells to mediate T cell and DC infiltration and differentiation. We first observed a robust release of type I IFN chemokines CXCL10 and (to a lesser extent) CCL20 from HT29-NLRC4 cells versus mock control pEx cells, along with myeloid growth factors M-CSF and GM-CSF, independently of exogenously added IFN-γ (Figure 6A). As a control, we confirmed the absence of inflammasome-dependent IL-1β and IL-18 release in response to NLRC4 expression and/or IFN-γ treatment. Consistent with our observation, CXCL10 was shown to direct the polarization of CD4^+^ T cells into potent effector IFN-γ^hi^IL-4^lo^ Th1 cells (15), and low transcript levels of *CXCL10* in CRC patients are associated with poor prognosis (16). CCL20 is involved in DC homing to gut-associated lymphoid tissue (17). Next, a broader untargeted secretomics analysis revealed additional type I IFN chemokines being released by NLRC4-expressing cells such as CXCL1, CXCL6, CXCL9, inflammatory cytokines (TNF, LIF, and IL-8), and growth factors involved in immune cell proliferation (TGF-β1, FGF-19, SCF, VEGFA, and TGF-α) (Figure 6A). To explore the capability of tumor-cell-derived NLRC4 expression to directly mediate DC maturation for T cell activation, we cocultured HT29-NLRC4 cells with freshly isolated human primary DCs from human blood. Cocultures of HT29-NLRC4 cells with DCs induced a significantly higher release of IL-12 compared with pEx control cells in the presence of LPS priming (while being maintained at same background level with monocultures of HT29-NLRC4 cells, pEx control cells, or DCs; in the presence or not of LPS) (Figure 6B). These results were confirmed at various LPS concentrations (Figure 6B). We also identified IFN-γ and IL-1β as following the same pattern as IL-12 (Figure 6C), in contrast with IL-2 and IL-10, used as controls (Supplemental Figure 17A). Broader untargeted proteomics analysis of these cocultures also identified the Th1-polarizing cytokine IL-18 and additional mediators involved in DC proliferation/differentiation (STAMBP, Flt3L, and 4E-BP1) being significantly released by DCs in the presence of NLRC4-expressing cells (Figure 6D). The plotted curves of the additional analytes captured by untargeted secretomics are shown in Supplemental Figure 17B. Consistent with these results, the combination of these multiple NLRC4-induced mediators secreted by DCs (GM-CSF, IFN-γ, and Flt3L) has been shown to robustly induce maturation of DCs for IL-12 production and Th1 stimulation (18). Since DC-mediated IL-12 and IL-18 secretion is critical in mediating T and NK cell cytotoxicity and Th1 polarization, including licensing cytotoxic CD8^+^ T cells in their antitumor activities (19, 20), we established here that NLRC4 expression in human cancer cells is sufficient to directly mediate the release of critical cytokines and immune mediators to drive DC maturation toward Th1 polarization in vitro. Hence, these mechanisms contribute to explaining the association between NLRC4 expression and DC/T cell tumor immune infiltration we observed in patient tumors, which correlates with improved survival.

### LPS specifically downregulates NLRC4 expression, but not that of other NLR family members, in human primary cells.

Progression to colon metastasis in patients has been suggested to be linked to the presence of higher LPS in mucosal tissues (21). To test whether LPS treatment affected expression of *NLRC4* in human immune cells, we stimulated primary monocytes and macrophages in vitro and found that *NLRC4* gene expression, along with that of its adaptor *NAIP* required for pathogen sensing, was dramatically reduced by LPS treatment in these cells (Supplemental Figure 18A). Strikingly, such decrease was not observed for other NLR family members *NLRP3* and *NLRP1* (Supplemental Figure 18B), nor for the inflammasome gene *CASP1* (Supplemental Figure 18C). The transcription factor IRF8 (which can control *NAIP* and *NLRC4* gene expression in mice) (22) was rather increased by LPS treatment (Supplemental Figure 18C), thereby suggesting another mechanism of repression independent of IRF8. Therefore, the presence of LPS-expressing bacteria may provide a mechanistic link between the loss of NLRC4 protein expression in CRC patient tissues and metastatic progression. Indeed, increasing evidence suggests that bacterial infection not only promotes carcinogenesis but also affects metastatic progression and organ selectivity through modification of the microenvironment at primary and secondary tumor sites (as reviewed in ref. 23).

## Discussion

Here we describe what we believe is a novel role for epithelial NLRC4 in cancer progression and invasiveness. Its expression in cancer cells predicts patient survival and modulates antitumoral immune responses in humans. We identified that tumor epithelial expression of NLRC4, rather than stromal, is critical to mediate immune protection through DC and CD4^+^ and CD8^+^ T cell immune infiltration into the tumor microenvironment. Mechanistically, we show that human epithelial NLRC4 expression can engage an immune transcriptional program combining type I IFN gene signaling and chemokine production, enabling the direct maturation of DCs toward a Th1 antitumor immune response in vitro. This underlying mechanism may explain our observed strong associations between NLRC4 tumor expression with DC2/DC3 and CD4^+^/CD8^+^ T cell infiltration into patient tumors for antigen presentation and tumor killing, respectively. Therefore, epithelial NLRC4 expression is transmitting critical information between the epithelial innate and adaptive immune responses against metastatic cancer progression, leading to improved patient survival.

The decreased expression of NLRC4 protein in the bulk tumor and its correlation to poor prognosis in CRC patients is consistent with a previous report (24). To note, upregulation in astrocytes was associated with poor prognosis in glioma patients (25). Our results show that in contrast with epithelial cell–expressed NLRC4, loss of IL-1β expression is not associated with disease prognosis. Hence, the impact of NLRC4 expression on the prognosis of CRC and protection against metastatic progression is unlikely to be mediated by downstream inflammasome pathway components alone. In comparison with mouse models, our results in patients are consistent with a previous study showing that *Nlrc4^–/–^* mice displayed increased colon tumorigenesis in the AOM/DSS model wherein tumors appeared aggressive, with invasion of tumor cells below the muscular mucosae (10). Another study using AOM/DSS showed no difference in *Nlrc4^–/–^* mice (26). Notably, a protumorigenic role of NLRC4 coming from the myeloid compartment has been described in mouse models of high-fat diet–induced CRC and breast cancer (27, 28), although the tumor-intrinsic role of NLRC4 was not investigated. NLRC4 is normally constitutively expressed in human intestinal epithelial cells along with pro-IL-18 (29), to cope with extracellular insults by sensing through NAIP the presence of infection and microbiome dysbiosis directly, thereby participating in maintaining the gut barrier integrity. However, when rendered hyperactivated by mendelian GOF mutations, NLRC4 induces a spectrum of clinical autoinflammatory syndromes characterized by severe enterocolitis and gut inflammation (5–7). These clinical features are clearly different from NLRP3 GOF–inducing CAPS diseases (cryopyrin-associated periodic syndrome). This observation suggests that activated NLRC4 triggers nonredundant immune mechanisms in the gut compared with other NLRs.

At the molecular level, we have identified what we believe are novel functions for human NLRC4 in triggering an immune cellular reprogramming, with type I IFN signaling and the production of a broad array of signaling molecules in vitro (chemokines, inflammatory cytokines, and growth factors) involved in the chemotaxis, proliferation, and activation of immune cells. In our study, expression of type I IFN genes induced by human NLRC4 can occur in the absence or presence of robust inflammasome activation. Therefore, both NLRC4 functions do not seem to be mutually exclusive, but may act in concert if needed. Since type I IFNs also induce *IL18* gene expression (29) and IL-18 upregulates MHCII expression in intestinal epithelial cells (30), we speculate that epithelial cell–intrinsic NLRC4 inflammasome activation may enhance local T cell activation through both type I IFN signaling and production of mature IL-18.

Consistent with our results in humans, a type I IFN signaling pathway was identified among the top upregulated gene networks in a patient harboring a dominant activating mutation in *NLRC4* (T337S), which leads to macrophage activation syndrome (5). Also consistent with our correlation of T cell infiltration in cancer patients with NLRC4 protein expression, an increase of gut intraepithelial lymphocytes was detected in the patient harboring the *NLRC4* activating mutation V341A, leading to enterocolitis autoinflammatory syndrome (6).

Important molecules mediated by human NLRC4 expression identified here include the CXCL10 chemokine and other family members, which are critically involved in the T cell–mediated antitumor immune response (15, 16, 31–34). We show that NLRC4 expression in human tumor cells is sufficient to directly promote DC maturation to secrete IL-12 and IFN-γ in vitro, along with the inflammatory cytokine IL-1β. DC-mediated IL-12 secretion is key in mediating T and NK cell cytotoxicity and Th1 polarization, including licensing cytotoxic CD8^+^ T cells in their antitumor activities in the context of anti–PD-1 immunotherapy (19, 20). Our findings indicate that this cytokine may, at least in part, mediate the downstream effects of NLRC4 expression in modulating the antitumor immune response against human CRC and possibly other epithelial cancers. Our results support a mechanism by which *NLRC4* expression drives DCs (mainly DC2, DC3, and DC4 subsets) and CD4^+^ and CD8^+^ T cell infiltration into patient tumors. DC2 and DC3 subsets express molecular features of antigen presentation, as shown previously by scRNA-seq, and promote CD4^+^ and CD8^+^ T cell activation/proliferation in cocultures (14). In human CRC, MSI^hi^ patients with MMRD tumors have been shown to have better antitumor immunity, with cytotoxic T cell infiltration and response to ICB (12). An enriched immune hub within MMRD tumors has been identified, composed of activated T cells and malignant cells expressing IFN-stimulating genes and CXCR3 ligands. We show here that higher *NLRC4* expression is also associated with MSI^hi^ CRC patient tumors. Putting these results into context with our observations, we can further refine this model and propose a critical role for epithelial cell–expressed human NLRC4 to trigger DC2/DC3 and T cell infiltration through type I IFN signaling and chemokine production to subsequently prime and activate T cells for tumor killing. As a result, NLRC4 expressed in the epithelium prevents progression to a more aggressive metastatic stage of CRC by contributing to this antitumor immune hub. Additionally, since MSI^hi^ status predicts better responses to ICB immunotherapies and NLRC4 associates with improved T cell infiltration, conferring better prognosis, we hypothesize that NLRC4 expression might provide a better response to ICB or broaden the scope of potential patient responders. Our observation that *NLRC4* expression is strongly associated mainly with CD4^+^ T cell infiltration and *CD4* gene expression more potently than *CD8A* in patient tumors indicates that NLRC4 might play a predominant role in the acquisition of CD4^+^ T cell cytotoxic function, previously demonstrated to be critical for mounting a productive antitumor immune response (35). This effect could be explained by the infiltration and maturation of tumor-antigen-presenting DC2 cells, as observed in melanoma patients (36).

The identity of damage-associated molecular patterns (DAMPS) or pathogen-associated molecular patterns (PAMPs) that activate human NLRC4 in epithelial tumor cells leading to the phenotypes described in this study warrants further investigation. In addition to its role in sensing bacterial pathogens, NLRC4 activation can be triggered by sterile DAMPs produced during metabolic dysregulation or expression of endogenous retrotransposons (3, 4). Since SINE RNAs robustly accumulate early during malignant cellular transformation (37) and de novo purine synthesis contributes to the proliferation of cancer cells (38), we speculate that these DAMPs could be sensed intrinsically by epithelial cell–derived NLRC4, leading to a robust immune response. Finally, we show that the loss of NLRC4 expression in patient tumor tissues may be due to the presence of LPS (expressed by gram-negative bacteria), which specifically abolished *NLRC4* expression at the transcriptional level but not that of other NLR family members in human primary cells. These results are consistent with previous findings demonstrating that LPS promotes metastatic progression in CRC through an NF-κB/Snail/HK3 signaling axis that potentiates glycolysis and increases migration and invasion (21). Therefore, the loss of *NLRC4* transcripts might be mediated through this axis (since *IRF8* remains unchanged) and might be explained by the presence of an LPS-enriched microbiome in patients.

In summary, we propose what we believe is a novel mechanism of immune protection against tumors in humans mediated by the expression of epithelial NLRC4. Our combined analysis using genetic editing, RNA-seq, and proteomics enabled us to identify the pathway by which NLRC4 expression associates with enhanced DC profiles and T cell responses in cancer patients, controlling the evolution of the disease and improving the survival of patients. This work sheds light on epithelial NLRC4 in providing critical priming signals to improve DC and T cell immune response in humans, thereby eventually sensitizing the tumor to current T cell–centric ICB therapies. These results might lay the foundation for novel therapeutic opportunities in CRC and other epithelial cancer types by extension, including lung and skin melanoma.

## Methods

### Sex as a biological variable

Both sexes were involved, and sex was not considered as a biological variable.

### TMAs from the Bergonié Cancer Institute

One hundred and four patients treated for primary CRC at the Bergonié Cancer Institute between May 2008 and January 2013 were enrolled. Histology samples were obtained from surgery, and tissues were fixed in formalin and then paraffin embedded (FFPE). Clinical characteristics were collected from patient medical charts with special focus on age, sex, date of diagnosis, and tumor node metastasis (TNM) stage. Histological type was determined, as well as tumor grade and mutational status. Tissue cores with a diameter of 0.6 mm were removed from FFPE blocks and arrayed on a recipient paraffin block using a tissue arrayer (Beecher Instruments Tissue Arrayer) at the Molecular Biology Department of the Bergonié Cancer Institute. Each tumor sample was punched in triplicate, along with 2 cores of matched normal mucosal tissue punched far away from the tumor. Sections of the array were cut at 5 μm and placed on glass slides.

### TMAs from the commercial USBiomax cohort

In the CO2161 TMA (USBiomax Inc.), there were 204 colon adenocarcinoma, 4 signet-ring cell carcinoma, and 8 unmatched normal colon tissues, with 1 core for each tumor case. Adenocarcinomas were from pathologic stage I (*n* = 22), stage II (*n* = 128), stage III (*n* = 47), and stage IV (*n* = 11) (Supplemental Table 1). Slides were treated similarly to the Bergonié TMA slides, and were stained only for NLRC4 and cytokeratin.

### Immunofluorescent tissue staining

Immunofluorescence analysis was performed on 5-μm FFPE TMA sections mounted on charged slides. Tissues were deparaffinized in xylene and rehydrated in a series of ethanol baths. Heat-induced proteolytic epitope retrieval was done in target retrieval buffer (DAKO pH 6.0 S236984 or pH 9.0 S236784, Agilent) according to the primary antibody using a microwave oven for 20 minutes. Slides were then blocked using 5% BSA for 10 minutes. Primary antibodies (see below) were incubated in DAKO antibody diluent (S202230, Agilent). Secondary antibodies (goat anti-rabbit–Alexa Fluor 594, goat anti-mouse–Alexa Fluor 488, and donkey anti-goat–Alexa Fluor 594; Thermo Fisher Scientific) were diluted (1:400) in antibody diluent. Sections were washed with PBS and incubated with DAPI (2 μg/mL) for 10 minutes. Finally, slides were mounted with Fluoromount-G and stored at 4°C. Primary antibodies were against the following proteins: NLRC4 (Abcam, ab115537), caspase-1 (p10) (LS Bio, LS-C312683), IL-1β (Cell Signaling Technology, 3A6), IL-18 (Sigma-Aldrich, HPA003980); and pan-cytokeratin to specifically stain epithelial cells (clone AE1/AE3, Dako, M3515). Before use on TMA slides, we assessed the antibodies’ specificity by Western blotting or immunofluorescence assay on FFPE cell lines.

### Image acquisition from TMAs

#### Image handling.

Images of the TMAs were generated using the NanoZoomer 2.0-HT slide scanner from Hamamatsu. Custom software was designed (QuantaCell) to perform the processing adapted to the study.

#### Cytokeratin detection.

Epithelium was detected using a simple threshold intensity on the cytokeratin channel followed by a morphological closing operation to fill small holes in the segmented region. The obtained region could be used to distinguish epithelial structures from the rest on the image. It also could be transferred to a consecutive slice to reveal the position of the epithelium in the consecutive slides.

#### Cell detection.

The nuclei DAPI channel was used to drive cell segmentation. Nuclei were detected using band-pass followed by thresholding above the background. Nuclei clusters were split using a watershed strategy to obtain well-separated nuclei segmentation. Cell segmentation was obtained by defining rings around nuclei. Rings of fixed width defined the cytosolic area around nuclei.

#### Feature measurements.

For each cell, intensity features were measured from fluorescence channels using maximum or median or average operators. Measurements were extracted for the different subcellular compartments (cells, nuclei, and cytosol). Cell statistics were stored as csv files for further analysis.

#### Core alignment.

To perform correlative analysis between the different channels, a method based on image registration was developed and used. For each core, image registration based on the DAPI channel was applied. The DAPI channel was used because it is the channel that is the most conserved from one slide to another. The image registration consists in finding the best rigid transform (translation + rotation) that allows fitting the first image into the second. This best transform was applied to all channels of the first slide. A new multiplexed image was obtained, combining all channels of the first slide and all channels of the second slide. This multiplexed image was used to apply cytokeratin mask detection to consecutive images.

#### Cytokeratin region transfer.

To be able to transfer the epithelial region to a consecutive slide, the nuclei channel of a core image was registered to the nuclei channel in the consecutive slide. Nuclei repartition and density contains enough information to estimate the rotation and translation needed to register the first image on the second one. The estimated rotation and translation were applied to the full core image and the epithelial region mask, thereby precisely aligning the epithelial region mask with the consecutive slice.

#### Cytokeratin mask detection.

The cytokeratin mask was obtained by processing the cytokeratin channel using direct thresholding followed by morph math operations (opening and closing). This mask was used as a channel such as a feature to express whether the cell is in or out the cytokeratin mask.

#### Cell positivity.

Cell positivity was obtained by placing one or several thresholds on selected features. For example, we counted the number of cells that are positive for a certain biomarker and inside the cytokeratin mask. The positive cells were displayed on the screen such that the user can control and fine-tune the threshold. After staining, slides were then digitalized with the Hamamatsu NanoZoomer 2.0-HT scanner in collaboration with the Bordeaux Imaging Center. Imaging acquisition and fluorescence quantification were performed by QuantaCell.

After computer-assisted image calibration, immunofluorescence quantification was obtained by measuring the fluorescence of each pixel in a DAPI-positive cell, calculating the median of all pixels in each cell, and then assessing the mean of median intensity of all cells for each spot. Studied cells were identified with DAPI staining and epithelial cells (in normal and tumor tissues) with a cytokeratin mask (Supplemental Figure 1). Indeed, quantification of inflammasome expression was done by applying a cytokeratin mask on each spot, to measure fluorescence of inflammasome markers only in cytokeratin-positive epithelial cells. Images were reviewed with NDP.view 2 (Hamamatsu).

### Immunohistochemistry

Immunohistochemical analysis was performed in all cases on 3-μm-thick serial sections from a representative FFPE block. We used antibodies against the following proteins: CD3 (clone 2GV6, prediluted, Roche Diagnostics), CD8 (clone C8/144B, diluted 1:25, Dako), CD68 (clone PG-M1, diluted 1:50, Dako), and CD163 (clone 10D6, diluted 1:100, Leica Novocastra Laboratories). In 32 cases, heterologous differentiation was suspected and additional antibodies against desmin (clone D33, diluted 1:100, Dako), h-caldesmon (clone h-CD, diluted 1:50, Dako), and myogenin (clone LO26, diluted 1:20, Novocastra) were used. After microwave oven heating (20 minutes in 0.1 M citrate buffer at pH 6), sections were incubated with biotinylated link antibody, and then with peroxidase-labeled streptavidin (LSAB + Kit; Dako), and finally with diaminobenzidine solution (DAB; Dako). Samples without the specific primary antibody were used as negative controls. Levels of tumor infiltration by immune cells was assessed by an expert pathologist based on cell morphology and organization. Then, levels of tumor infiltration specifically by CD3^+^ and CD8^+^ cells (T cells), or CD68^+^ and CD163^+^ cells (macrophages) were assessed as low, moderate, or high.

### Cloning procedure and control

cDNA encoding *NLRC4* was amplified by RT-PCR and directly ligated into a lentiviral vector. The mCherry vector was provided by GeneCopoeia (pEx-T3678-Lv130) and *NLRC4* was already ligated in the vector. In this vector, *NLRC4* is fused at its C-terminus with the red fluorescent protein mCherry. Lentiviral particles were produced by transient transfection of packaging HEK 293T (ATCC) cells and viral titers were determined by ELISA of p24. Transductions of HT29 (ATCC) were performed using lentiviral particles produced by transient transfection of HEK 293T cells. Different MOIs were used in RPMI 1640 medium with 8% FBS and glutamine. Purity was assessed by FACS and cells from correct MOIs were sorted by flow cytometry (BD FACSAria). *NLRC4* expression was assessed by RT-qPCR and Western blotting (primary antibody Abcam, ab115537, diluted 1:1000; fluorescent secondary antibody diluted 1:4000, IRDye).

### Proliferation assay

Cell division was assessed using carboxyfluorescein diacetate succinimidyl ester (CFSE) dye (Life Technologies, V12883). Transduced cells with NLRC4-mCherry or mock-mCherry were trypsinized, spun down, and resuspended at 1 × 10^6^ cells/mL in PBS containing CFSE at a final concentration of 1.5 μM. Cells were stained for 10 minutes at 37°C and then washed in hot sterile PBS. Cells were centrifuged and resuspended in complete medium at a concentration of 1 × 10^6^ cells/mL for 30 minutes at 37°C. Cells were then washed twice in medium and 20,000 cells/well were seeded in 24-well plates. Cell division was analyzed every 24 hours by detecting fluorescence by flow cytometry, until 7 days.

### Scratch wound healing assay

Cells were seeded at 400,000 (HT29) or 450,000 (SW620) cells/well in a 24-well culture plate. Cells were cultured until reaching 80% confluence as a monolayer. After 24 hours of growth, a 200 μL pipette tip was used to form a wound. Each well was gently washed twice with medium to remove detached cells, and cells were cultured for a further 24 hours. A microscope at ×4 magnification was used to acquire pictures and the scratch surface was assessed at each time point using ImageJ software (NIH).

### Differential gene expression by RNA-seq analysis

RNA-seq sample analysis was performed using Array Studio software version 10.0.1.81 (QIAGEN), the OSA aligner, and the OmicsoftGene20130723 gene model (39). Aligned count matrixes were used as input into a DESeq2 workflow to measure differential gene expression (40). Genes with an absolute log(fold change) of greater than 2 and an adjusted *P* value of less than 0.05 between 2 conditions were considered significant. Significant genes were used as input to the pathfinder R package to measure altered KEGG pathways (41, 42).

### Bioinformatic analysis of public data sets: profiling of immune cell infiltrates, survival, and correlation of gene expression

Immune cell type gene signatures were from CIBERSORT (43). DC subset gene signatures were from scRNA-seq study of human blood DCs (14), The Cancer Immunome Atlas (TCIA; https://tcia.at/ Accessed October, 2019.) (44), and TIMER (45). Type I IFN–regulated genes were those reported on the QIAGEN Human Type I Interferon Response PCR Array and whose upregulation by type I IFN was confirmed by at least 2 studies in the Interferome database (https://interferome.org/interferome/home.jspx Accessed January, 2017.). Pearson’s correlation analysis was performed for each pair of gene versus immune cell type, gene versus gene for their expressions in COAD, READ, SKCM, LUSC, and LUAD from TCGA transcriptomic data. The expression value of the cell type signature is represented by the median value of genes in the specific gene set. TIMER analysis (45) was conducted on the corresponding website. The ggpubr and ggplot2 packages in R (https://cran.r-project.org/web/packages/ggpubr/index.html; https://github.com/tidyverse/ggplot2) were used for statistical calculations and visualization. Comparisons of NLRC4 between tumor samples and related normal samples were conducted in “TCGA land” by OmicSoft. Survival analysis was performed in the ClinicalOutcome data set compiled by OmicSoft (http://www.arrayserver.com/wiki/index.php?title=Introduction_to_ClinicalOutcome_Land_Content Accessed October, 2019.). ClinicalOutcome contains human gene expression profiles associated with key clinical features and outcomes like “Survival,” etc., from the NCBI Gene Expression Omnibus (GEO).

### Intestinal APC^–/–^ in vivo mouse model

#### Mice.

*Apc^Min/+^* mice (strain 002020) were obtained from The Jackson Laboratory and bred in house. Mice were sacrificed at 3 or 6 months of age. Small intestine and colon were removed from animals, flushed with cold PBS supplemented with penicillin and streptomycin, and cut longitudinally for polyp enumeration.

#### H&E staining and immunofluorescence.

Small intestines were fixed and subsequently paraffin-embedded. H&E sections were scanned using a ScanScope XT digital scanner (Aperio Technologies). For immunofluorescence, slides were deparaffinized by washing in xylene. Antigen retrieval was performed, and slides were permeabilized with 0.25% Triton X-100 in PBS and washed in PBS with 0.05% Tween 20. Slides were then blocked (10% FBS, 3% BSA [Bioshop, ALB001]) for 30 minutes at 37°C and tissues were incubated with primary antibodies in PBS containing 3% BSA, overnight at room temperature. Primary and secondary antibodies were diluted in 3% BSA in PBS. Primary antibodies against NLRC4 (Novus, NBP1-78980) and cytokeratin (AE1/AE3) (Dako, M3515) were used. Tissues were mounted with cover slips and analyzed on a Zeiss Axioskop upright wide-field microscope (20×/0.5 or 40×/0.75 Plan-Neofluar objectives) equipped with a high-resolution monochromatic AxioCam HRm camera and driven by AxioVision version 4.9.1 (Carl Zeiss Microscopy). ImageJ 1.46 (NIH) was used for processing of entire images before cropping to emphasize the main point of the image when appropriate; processing was limited to background subtraction, brightness/contrast adjustments, and pseudocolor addition to facilitate the visualization/interpretation of the results.

### mRNA transfection of human primary monocytes

Polyadenylated CleanCap in vitro–transcribed (IVT) mRNA fully substituted with 5-methoxyuridine encoding EGFP, NLRC4 (V341A), or NLRP3 (R260W) was purchased as a custom order from Trilink Biotechnologies. Nine picomoles of each IVT mRNA was electroporated into peripheral primary human monocytes using the 4D-Nucleofector System (Lonza) with the P3 Primary Cell 4D-NucleofectorTM X Kit L (program EA-100). Twenty-four hours after electroporation, supernatants were harvested and assayed for caspase-1 activity using Caspase-Glo 1 (Promega) and IL-18 levels were measured using the MSD Multi-Spot assay system. Total RNA was isolated from the cellular fraction of triplicate-pooled samples using the RNeasy kit (QIAGEN, 74106) and subjected to cDNA synthesis using the Iscript cDNA synthesis kit (Bio-Rad, 1708891). Quantitative PCR was performed on cDNA samples using TaqMan primer/probes purchased from Thermo Fisher Scientific. Data were normalized to the levels of the housekeeping gene *ACTB* and plotted as fold change versus control transfection.

### Cocultures of human primary DCs and maturation, and secretomics analysis

HT29 CRC cells containing either pEx (ref. pEx-T3678-Lv130) empty vector or pEx-NLRC4 were cultured according to previously stated conditions (Gibco RPMI 1640, 8% FBS, 200 mM GlutaMax [Gibco, 35050061]). HT29 cells were grown to 60%–70% confluence and then trypsinized (Gibco, 25300120) and counted using trypan blue 0.4% (VWR, K940) for dead cell exclusion. HT29 pEx and HT29-NLRC4 cells were plated on 96-well plates (VWR, 10861-562) at 35,000 cells per well. Buffy coats from healthy donors at the Stanford Blood Center were collected and PBMCs were isolated using density-gradient centrifugation (Ficoll-Paque, GE Healthcare, 17-1440-02) and enriched for DCs with STEMCELL Technologies EasySep Human Pan-DC Pre-enrichment kit (catalog 19251; EasySep Magnet, 18000; EasyEights, 18103; EasySep Buffer, 20144). DCs were counted and plated at a 1:1.2 ratio (35,000 HT29s:43,000 DCs) on top of HT29s in preplated 96-well plates. LPS (Invitrogen, 00-4976-93) was added to cocultures at 0, 0.1, 0.316, 1, 2, 3.16, 10, and 31.62 μg/mL concentrations in either duplicate or triplicate. Cocultures continued for 24 hours and cell supernatants were centrifuged, placed in fresh tubes, and frozen at –20°C. Quantikine IL-12 p70 ELISA (R&D Systems, D1200) was performed according to the manufacturer’s instructions to assess DC priming. In parallel, the same cell supernatants were submitted for secretomics analysis by Olink Proteomics. Samples were prepared for and analyzed using the Inflammation I multiplex according to the manufacturer’s instructions (Olink Biosciences). To calculate the differential protein expression, a linear model was fitted with the log_2_-normalized protein expression levels as the dependent variable. For these calculations, we only used LPS-induced samples. *P* values were adjusted for multiple testing using the Benjamini-Hochberg method (46).

### Cellular models of NLRC4 (T337S) mutant expression

Primary human monocyte-derived macrophages and NLRC4-transduced THP1 cells (ATCC) were generated as in Canna et al. (5). Briefly, primary macrophages were generated from CD14-selected peripheral blood monocytes by culturing in M-CSF for 7 days. Stably transduced THP1 cells were stimulated with 10 ng/mL PMA for up to 72 hours. Cells were lysed in TRIzol, RNA isolated, and RNA integrity analyzed with the Agilent 2200 Tapestation. mRNA purification and fragmentation, cDNA synthesis, and target amplification were performed with an Illumina TruSeq RNA Sample Preparation kit. Pooled cDNA libraries were sequenced on an Illumina HiSeq 2000, mapped to XXXX, gene track XXXX, quantified using CLC Main Workbench software (v22, QIAGEN), and expressed as transcript per million (TPM). Heatmaps were generated using Morpheus (https://software.broadinstitute.org/morpheus). Gene set enrichment analysis (GSEA) was performed on untrimmed TPMs comparing the T337S mutant– versus WT–transduced THP1 cells at 24 hours using GSEA v4.1 (https://www.gsea-msigdb.org/gsea/index.jsp), with the following parameters: permutations = 1000; permutation type = gene_set, Enrichment statistic = weighted, and Ranking metric = Signal2Noise.

### Monocyte or monocyte-derived macrophage differentiation and stimulation

Pan human monocytes were isolated from peripheral blood using column-based isolation according to the manufacturer’s protocol (Miltenyi Biotec, 130-021-221). Isolated cells were then cultured in X-Vivo 15 media supplemented with 10 ng/mL MCSF (R&D Systems, 216-MCC-010) for 5 days, prior to stimulating them or freshly thawed monocytes from the same donor with 1 μg/mL LPS (Invivogen, tlrl-peklps) for 4 hours. RNA was then isolated and qPCR performed on cells according to the procedures described above. In experiments with needle-tox (*Bacillus* *thailandensis* T3SS needle protein [Needle] mixed with Protective Antigen [ListLabs] at a ratio of 1:8), isolated monocytes were rested overnight in media and stimulated the next day with either 0.1 ng/mL or 1 ng/mL needle-tox for the indicated periods of time. Cell supernatants were collected for assessment of caspase-1 activity according to the manufacturer’s protocol (Promega) or IL-18 secretion was measured by MSD. RNA was then isolated and qPCR performed in cell samples according to the procedures described above.

### Statistics

For analysis of patient tissue expression, clinical and biological measurements are expressed as mean (SD) or median (range) for continuous variables, or as a number (percent) for categorical variables. In the box-and-whisker plots, the whiskers indicate the minimal and maximal values, showing all points. The bounds of boxes indicate the width of distribution of points proportionate to the number of points at that *y* value. The lines within the boxes represent the *y* mean value of all data points. The comparison between quantitative and qualitative variables was done using a 2-tailed Student’s *t* test (parametric) or the Mann-Whitney *U* test (nonparametric). The comparison between qualitative variables was done using a χ^2^ (parametric) or a Fisher’s exact test (nonparametric). Variance analysis was done using a 1-way ANOVA (parametric) or Kruskal-Wallis (nonparametric) test. A Cox model was applied for survival analysis. Patients were stratified based on high versus low expression of markers according to cutoffs either predetermined by receiver-operating characteristics (ROC) curves (for NLRC4 and IL-18), or by the median fluorescence intensity (MFI) (for IL-1β). A logistic regression analysis was performed to evaluate the association between NLRC4, IL-18, or IL-1β levels and death within the tumoral tissue and healthy one. ROC curves and area under the curve were computed to assess the impact of marker expression levels on predicting death. Statistical significance was set at a *P* value of less than 0.05. Data analysis was performed using STATA software (StataCorp). ROC curves and threshold determination obtained for NLRC4 and IL-18 are shown in Supplemental Figure 5. However, for IL-1β, sufficient sensitivity and specificity could not be reached to use the determined cutoff and the MFI was used instead to stratify patients. Other statistical analyses are described in figure legends for other experiments.

### Study approval

Samples were collected in accordance with French legislation and ethical codes under a protocol approved by the ethical committee of the Bergonié Institute (Bordeaux, France), and all patients gave their written consent for the use of their biological samples for research purposes. Animal experiments were performed according to the guidelines of the animal ethics committee of McGill University.

### Data availability

RNA-seq data sets are deposited in the NCBI GEO (accession GSE243588). All raw data are provided in the Supporting Data Values file.

## Author contributions

CD, SC, DF, and BF conceptualized the study. CD, SC, HL, JMB, CC, AN, LL, MFV, EEL, LS, SB, IM, JDC, FL, MD, VV, SN, VP, CR, and VR developed the methodology. CD, SC, HL, JMB, CC, AN, ERR, LS, SB, LL, MFV, IM, JDC, FL, MD, VV, SN, VP, CP, KN, CR, VR, PB, JDM, MS, SWC, DF, and BF conducted experiments. PB, JDM, MS, SWC, DF, and BF acquired funding. DF and BF supervised the project. CD, SC, HL, MS, SWC, DF, and BF wrote, reviewed, and edited the manuscript.

## Supplementary Material

Supplemental data

Unedited blot and gel images

Supporting data values

## Version 1 04/23/2024

In-Press Preview

## Version 2 06/03/2024

Electronic publication
