# Supplementary material for: Human NLRC4 expression promotes cancer survival and associates with type I interferon signaling and immune infiltration
Source: J Clin Invest. 2024 Apr 23;134(11):e166085. doi: 10.1172/JCI166085 (PMC11142746; doi:10.1172/JCI166085)
Supplement: Supplemental data [file jci-134-166085-s232.pdf]

Supplemental information

Human NLRC4 promotes cancer survival and is associated to Type-I Interferon signaling and immune infiltration

Domblides et al.

A

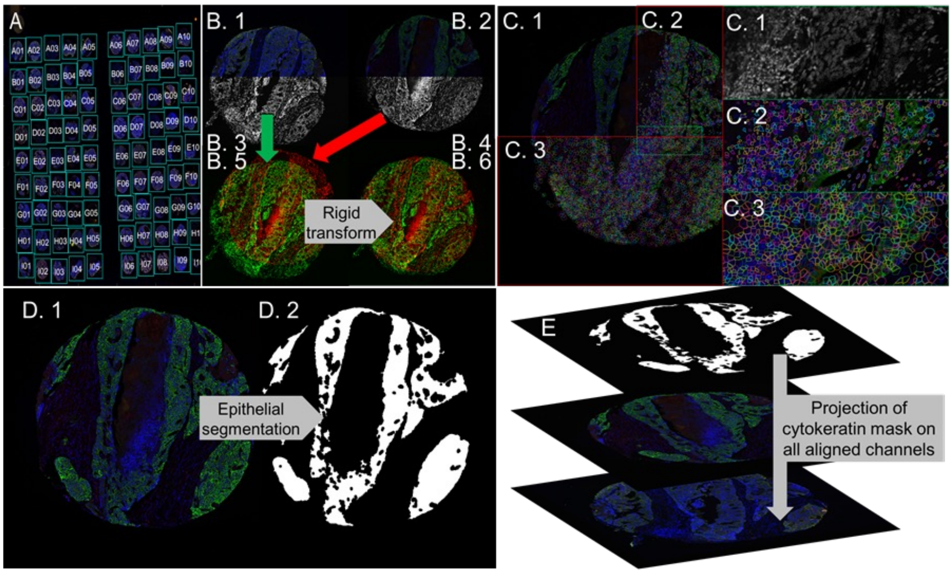

A
B. 1
B. 2
B. 3
B. 5
B. 4
B. 6
Rigid transform
C. 1
C. 2
C. 3
D. 1
D. 2
Epithelial segmentation
E
Projection of cytokeratin mask on all aligned channels

B

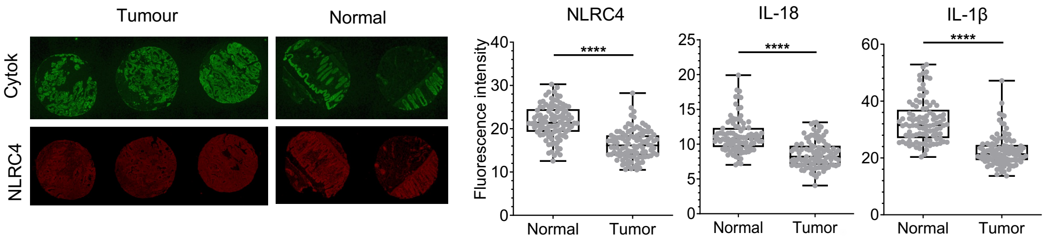

Tumour
Normal
Cytok
NLRC4
NLRC4
IL-18
IL-1β
Fluorescence intensity
****
Normal
Tumor

C

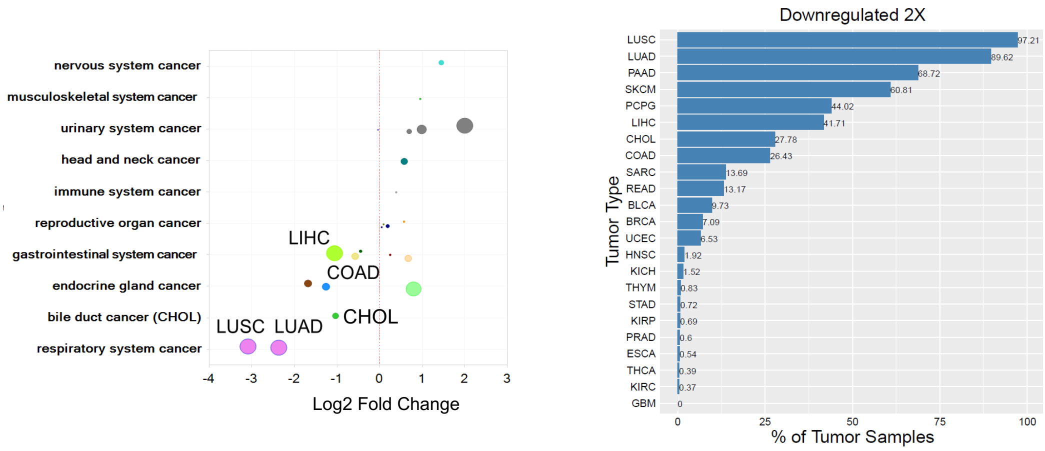

nervous system cancer
musculoskeletal system cancer
urinary system cancer
head and neck cancer
immune system cancer
reproductive organ cancer
gastrointestinal system cancer
endocrine gland cancer
bile duct cancer (CHOL)
respiratory system cancer
LIHC
COAD
CHOL
LUSC
LUAD
Log2 Fold Change
Downregulated 2X
Tumor Type
% of Tumor Samples
LUSC 97.21
LUAD 89.62
PAAD 68.72
SKCM 60.81
PCPG 44.02
LIHC 41.71
CHOL 27.78
COAD 26.43
SARC 13.69
READ 13.17
BLCA 9.73
BRCA 7.09
UCEC 6.53
HNSC 1.92
KICH 1.52
THYM 0.83
STAD 0.72
KIRP 0.69
PRAD 0.6
ESCA 0.54
THCA 0.39
KIRC 0.37
GBM 0

Supplemental Figure 1: High-Throughput tissue imaging of colorectal cancer patients shows loss of intratumoral NLRC4 expression. (A) High-throughput tissue image processing used to monitor the protein expression of NLRC4 and cytokines from patient tissue samples. Histology slides were stained with antibodies against NLRC4 or cytokines IL-1β, IL-18, or cytokeratin for epithelial cellular staining and DAPI for nuclear staining (step 1). Slides were acquired with the Hamamatsu Nanozoomer 2.0HT scanner. Nuclear staining (DAPI positive) and cellular segmentation was performed (step 2). A cytokeratin mask was then created and superpimposed on inflammasome staining (step 3). Quantification of immunofluorescence intensity was obtained by measuring the mean of median fluorescence intensity of DAPI positive and cytokeratin positive cells on each spot. (B) To the top, tissue imaging by immunofluorescence of cytokeratin mask and NLRC4 protein expression in 2 normal and 3 tumor patient tissues. To the bottom, protein expression of epithelial NLRC4, IL-18 and IL-1β in normal and tumor cells within the Tissue-Microarrays of colorectal cancer patients obtained from the Bergonié Cancer Institute (n=104 patients). Expression of markers was measured based on the average fluorescence intensity in each spot of tissue. Fluorescence intensities obtained from 3 tumor spots and 2 normal spots were averaged for each patient. Each spot in the graph represents one patient. Paired t-test (parametric) or Wilcoxon matched-pairs (non-parametric) were used to evaluate the significance of the difference between normal and tumor tissue. ****$P<0.0001$. (C) Gene expression analysis of *NLRC4* from the TCGA patient cohort, showing dysregulation among various cancer types and tissues of origin. To the left, comparison of *NLRC4* between tumor samples vs. related normal in each tumor type. Size of the dot represents the –log (adjusted pvalues). Tumor type is indicated in the Figure if *NLRC4* gene expression is downregulated with significant adjusted pvalue (< 0.05). To the right, percentage of samples with *NLRC4* having 2x down-regulation of gene expression in each tumor type.

Characteristics		N	%
Gender	Male	60	57.7
	Female	44	42.3
Median age (years [range])		71.7 [38.6 – 88.4]	
Stage	I	8	7.7
	II	36	34.6
	III	37	35.6
	IV	23	22.1
Grade	1	8	7.7
	1-2	9	8.7
	2	76	73.1
	3	8	7.7
Mutational status	KRAS	19	18.3
	BRAF	4	3.8
MMR status	MSS	88	84.6
	MSI	16	15.4
5-years overall survival		64.4%	

Supplemental Table 1: Clinical features of the colorectal cancer patient cohort used in the study. Clinical features of the Bergonié Institute cohort composed of 104 patients is described.

Characteristics		N	%
Gender	Male	118	56.7
	Female	90	43.3
Median age (years [range])		55.8 [24 – 82]	
Stage	I	22	10.6
	II	128	61.5
	III	47	22.6
	IV	11	5.3
Grade	1	73	38.2
	2	77	40.3
	3	41	21.5

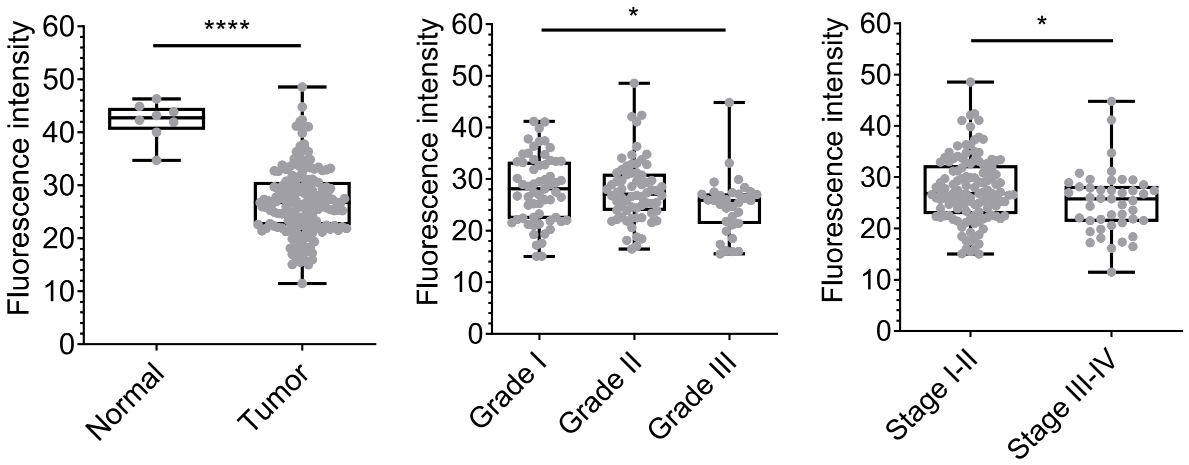

Supplemental Figure 2: Loss of tumor epithelial NLRC4 protein is associated with metastatic disease in CRC patients (validation cohort). (Top Table) clinical features of the validation cohort commercially available composed of 216 patients. Below to the left, protein expression of epithelial NLRC4 in normal and tumor cells within the Tissue-Microarrays of colorectal cancer patients obtained from the commercial validation cohort (n=216 patients). Expression of NLRC4 was measured based on the average fluorescence intensity in each spot of tissue. Each spot in the graph represents one patient. The validation cohort was obtained from USBiomax (see methods). *$p<0.05$; ****$p<0.0001$. In the middle, correlation of NLRC4 expression with tumor grade, showing an association between low expression and higher grade. To the right, protein expression of tumor epithelial NLRC4 in various CRC clinical Stages I-II, and III-IV.

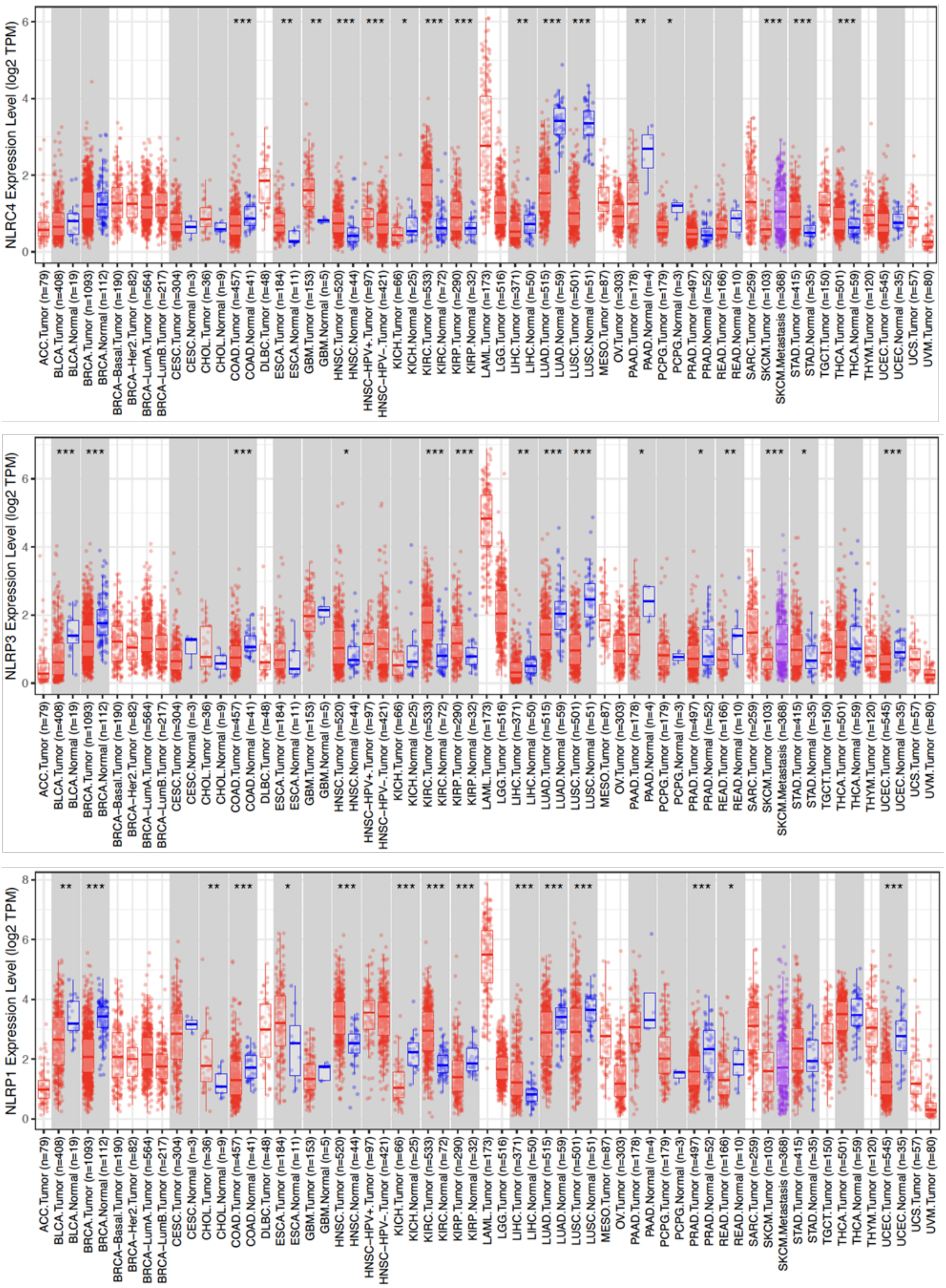

Supplemental Figure 3: Expressions of *NLRC4*, *NLRP3* and *NLRP1* in tumors vs. related normals in the TCGA patient dataset. Analysis was performed in TIMER (https://cistrome.shinyapps.io/timer/). P-value significant codes *p<0.05; **p<0.01; ***p<0.001.

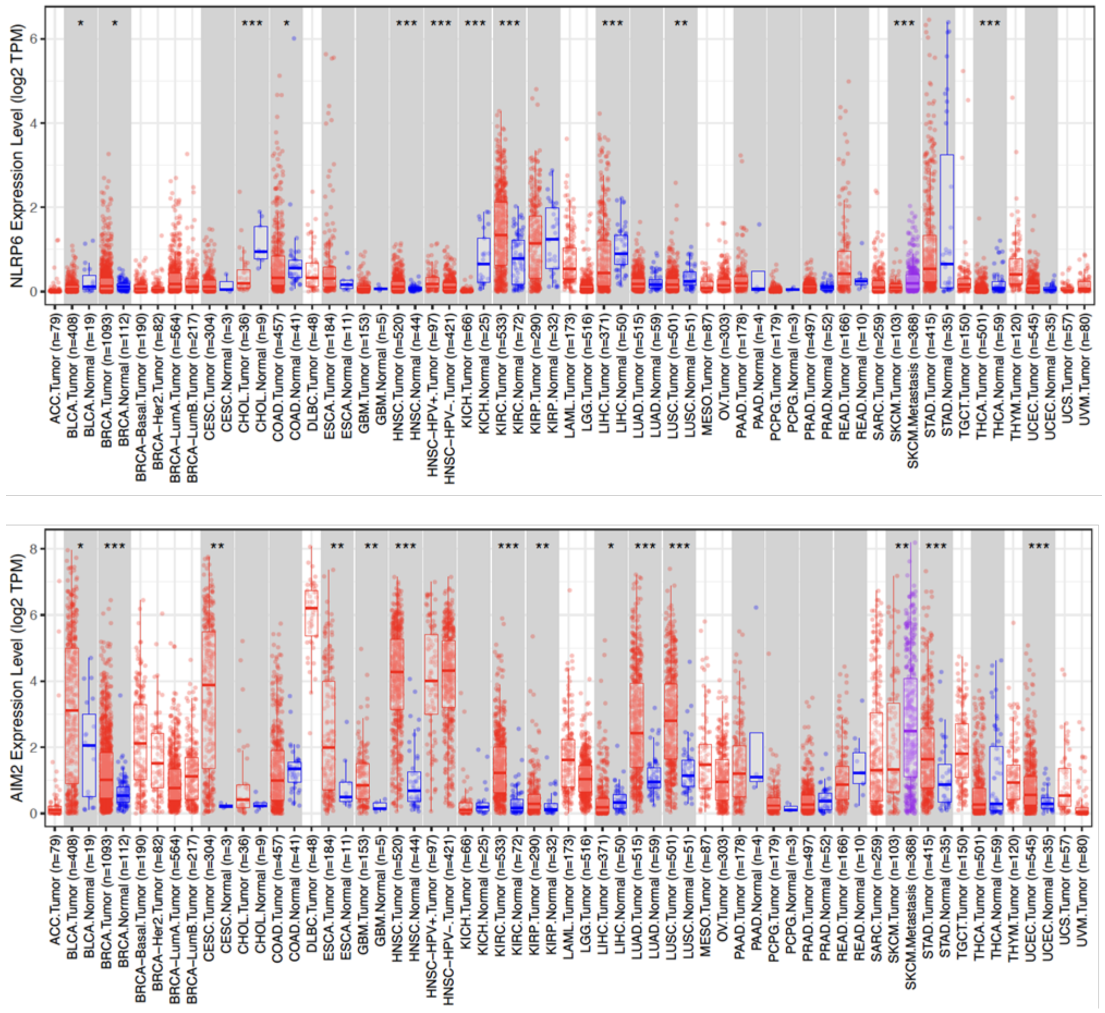

Supplemental Figure 4: Expressions of *NLRP6* and *AIM2* in tumors vs. related normals in the TCGA patient dataset. Analysis was performed in TIMER (https://cistrome.shinyapps.io/timer/). P-value significant codes *p<0.05; **p<0.01; ***p<0.001.

A NLRC4

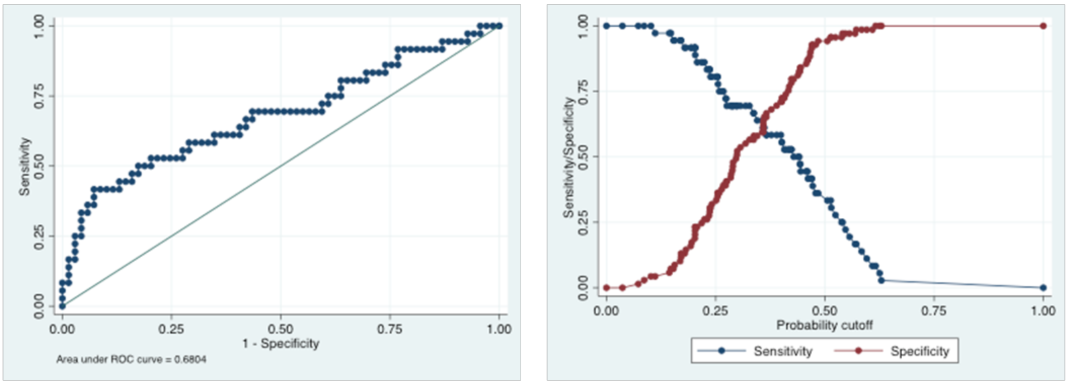

Threshold at 15.67565

	Death	Survival
NLRC4 +	22	25
NLRC4 -	14	44
Total	36	69

Se = 61.1% [43.5% - 76.9%]$_{95\%}$; Sp = 63.8% [51.3% - 75%]$_{95\%}$

B IL18

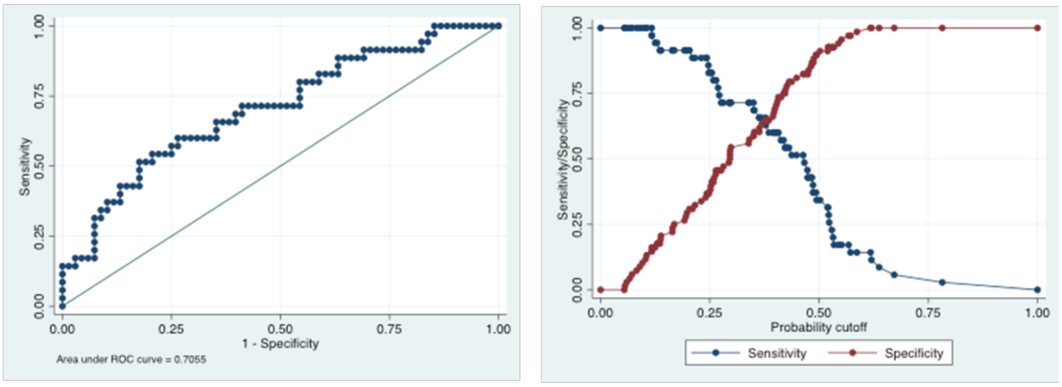

Threshold at 7.70055

	Death	Survival
IL18+	21	18
IL18-	14	50
Total	35	68

Se = 60% [42.1% - 76.1%]$_{95\%}$; Sp = 73.5% [61.4% - 83.5%]$_{95\%}$

Supplemental Figure 5: Generation of ROC curves for cut-off determinations of epithelial NLRC4 and IL-18 protein detection, sensitivity and specificity of each threshold. A logistic regression analysis was performed to evaluate the association between NLRC4 or IL-18 levels and death within the epithelial tumoral tissue. ROC curves and area under the curve were computed to assess the impact of marker expression levels for predicting death. Statistical significance was set at $p<0.05$.

ClinicalOutcome dataset

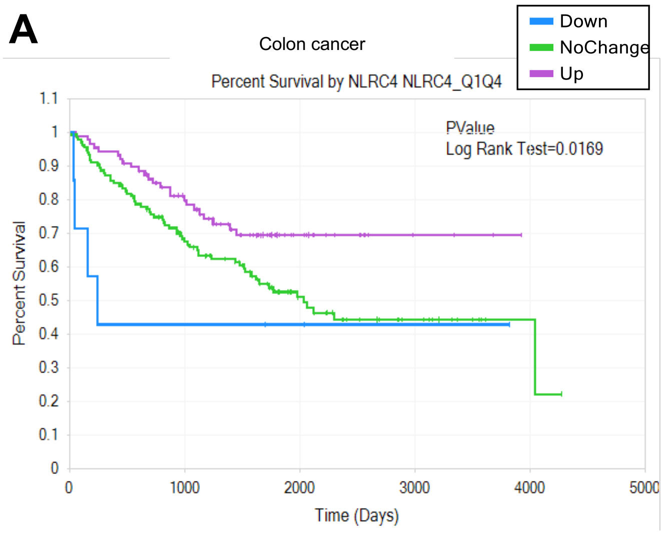

Cancer type	pvalue
Lung Cancers	0.0002 high vs low
Colon Cancer	0.0169 high vs low
Bladder Transitional Cell Carcinoma	0.0058 low vs high
Glioblastoma	0.0001 normal vs low
Brain Glioma	NS
Breast Cancer	NS
Ovarian cancers	NS
Leukemia cancers	NS
Astrocytoma cancers	data missing
B Cell Lymphoma cancers	data missing
Benign Neoplasm	data missing
Bone Osteosarcoma	data missing
Ewing Sarcoma	data missing
Fallopian Tube Cancer	data missing
Follicular Lymphoma	data missing
Gastrointestinal Stromal Tumor	data missing
Germ Cell Cancer	data missing
Head And Neck Squamous Cell Carcinoma	data missing
Hypopharynx Cancer	data missing
Melanoma	Only 1 group
Meningioma	Only 1 group
Mixed Germ Cell Cancer	data missing
Multiple Myeloma	data missing
Oral Squamous Cell Carcinoma	data missing
Peritoneum Cancer	data missing
Testicular Cell Tumor	data missing

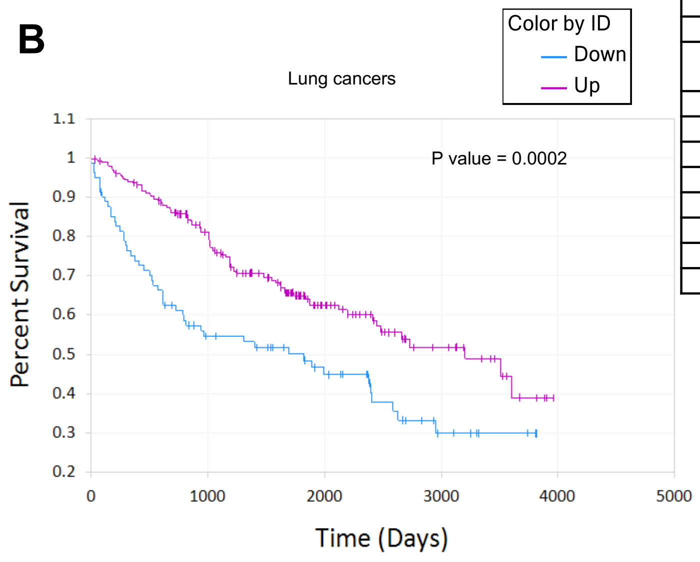

Supplemental Figure 6: Association between *NLRC4* gene expression (in tumor bulk) with overall survival of cancer patients. Data analyzed from the ClinicalOutcome patient dataset with Omicsoft. Colon cancer (A) or Lung cancer (B) patients were stratified based on transcript levels of *NLRC4* as up versus down expression (quartiles) in the tumor bulk. pvalues between up versus low expression are indicated. Table summarizes results for all cancer types available with directionality and associated pvalues; NS, not significant (>0,05).

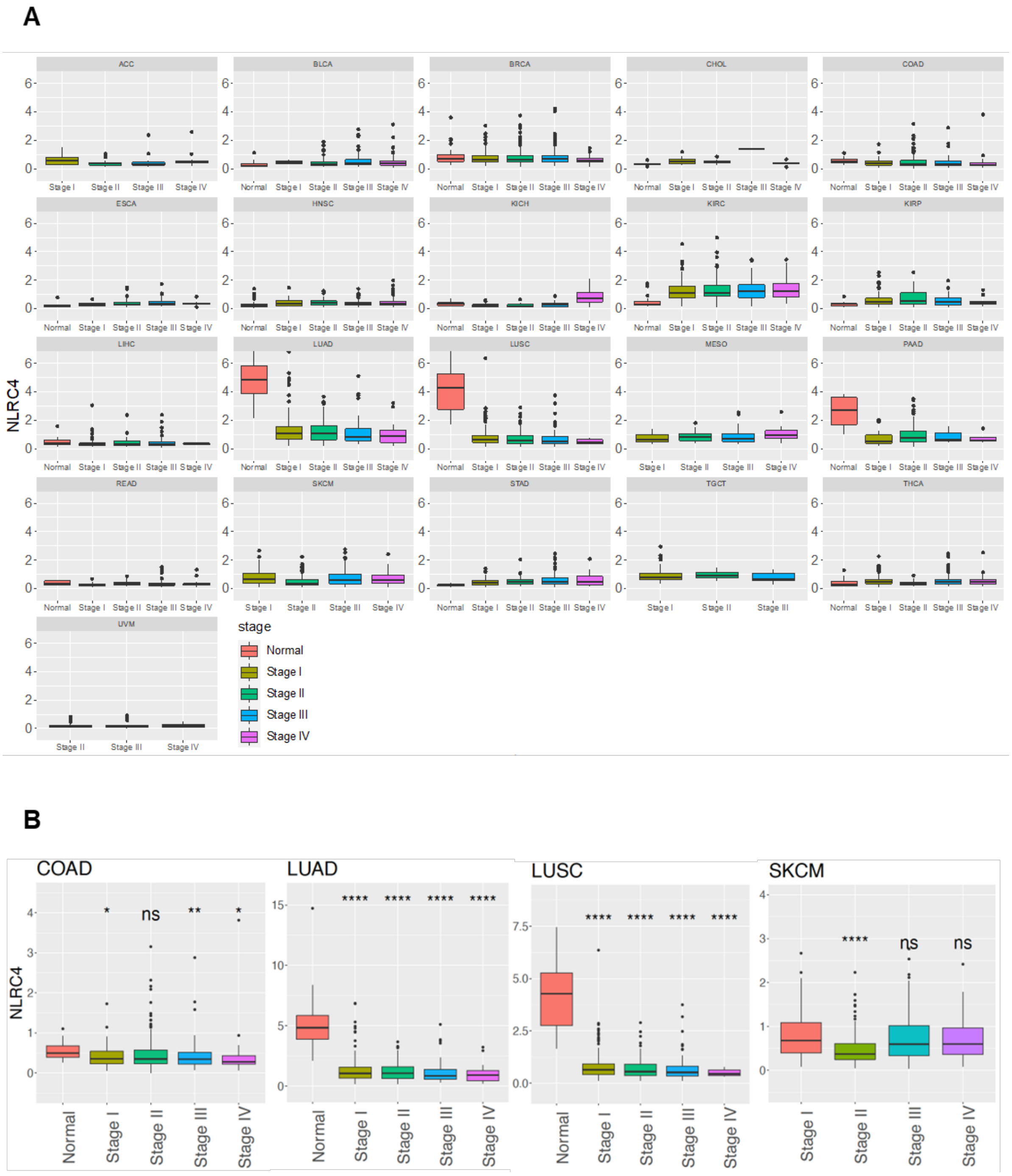

Supplemental Figure 7: *NLRC4* gene expression across tumor stages in the TCGA patient dataset. (A) Expression of *NLRC4* across tumor stages in each tumor type with Y axis at the same scale. (B) Expression of *NLRC4* across tumor stages in COAD, LUAD, LUSC and SKCM with Y axis at different scales. P-value significant codes *p<0.05; **p<0.01; ***p<0.001; ns not significant.

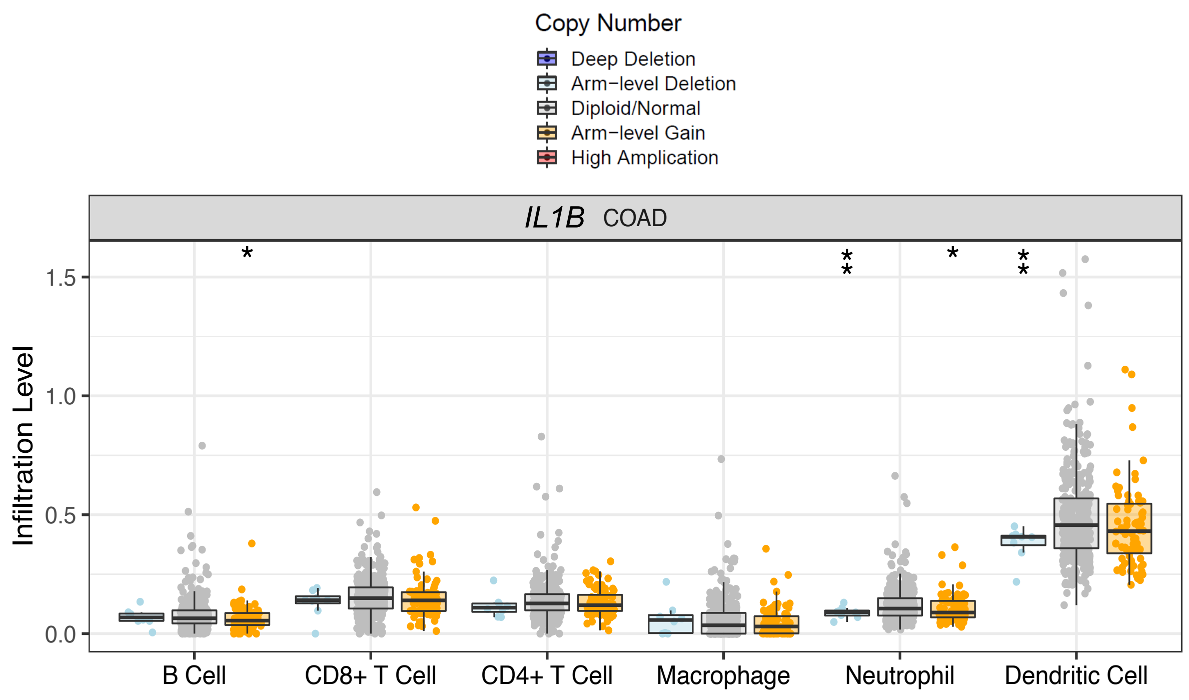

Supplemental Figure 8: Associations between *IL1B* somatic copy number alterations and composition of the tumor immune infiltrate. Data obtained from the TCGA cohort analysis and using the TIMER software. Box plots are presented to show the distributions of each immune subset at each copy number status in COAD cancer patients. The infiltration level for each category is compared with the normal using two-sided Wilcoxon rank sum test. *p<0.05; **p<0.01; ***p<0.001. COAD, colon adenocarcinoma.

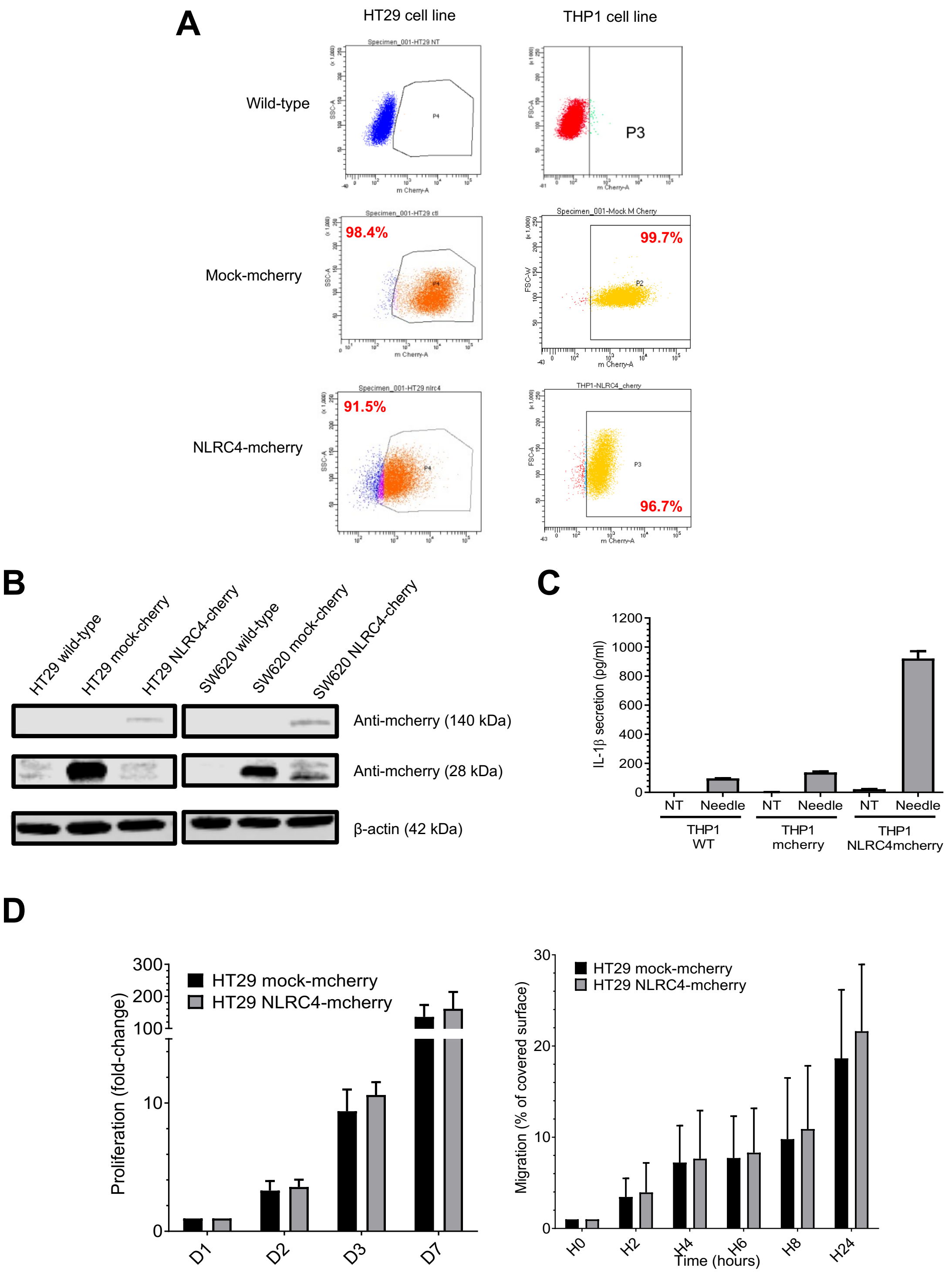
A
HT29 cell line
THP1 cell line
Wild-type
P3
Mock-mcherry
98.4%
99.7%
NLRC4-mcherry
91.5%
96.7%
B
HT29 wild-type
HT29 mock-cherry
HT29 NLRC4-cherry
SW620 wild-type
SW620 mock-cherry
SW620 NLRC4-cherry
Anti-mcherry (140 kDa)
Anti-mcherry (28 kDa)
β-actin (42 kDa)
C
IL-1β secretion (pg/ml)
NT
Needle
THP1 WT
THP1 mcherry
THP1 NLRC4mcherry
D
Proliferation (fold-change)
HT29 mock-mcherry
HT29 NLRC4-mcherry
D1
D2
D3
D7
Migration (% of covered surface)
Time (hours)
H0
H2
H4
H6
H8
H24

Supplemental Figure 9: THP1-NLRC4 stable cell lines. Stable cell lines were engineered using human NLRC4-expressing lentiviruses (Methods). NLRC4 is fused to the fluorescent tag mcherry at C-terminus, and expressed under CMV promoter. Mock-mcherry (empty vector) was used as a control, expressing only the fluorescent tag but not NLRC4. (A) After lentivirus infection at different MOIs, cells were sorted based on mcherry fluorescence and purity was assessed by flow cytometry as indicated. (B) NLRC4 protein expression was assessed by western-blot by using either anti-NLRC4 or anti-mcherry specific antibodies. (C) NLRC4mcherry expressed in stable cell line is functional and can induce inflammasome activation. THP-1-NLRC4mcherry or THP-1mcherry cell lines were treated with DOTAP+Needle to activate NLRC4, and IL-1β secretion was determined by ELISA from cell supernatants. Data as mean +/- SD (n=3 independent experiments)**.** (D) Proliferation assay performed on HT29-NLRC4 cell line or mock cell line using CFSE. Migration and invasion assays performed on HT29-NLRC4 cell line or mock cell line using scratch test. Data as mean +/- SD (n=3 independent experiments)

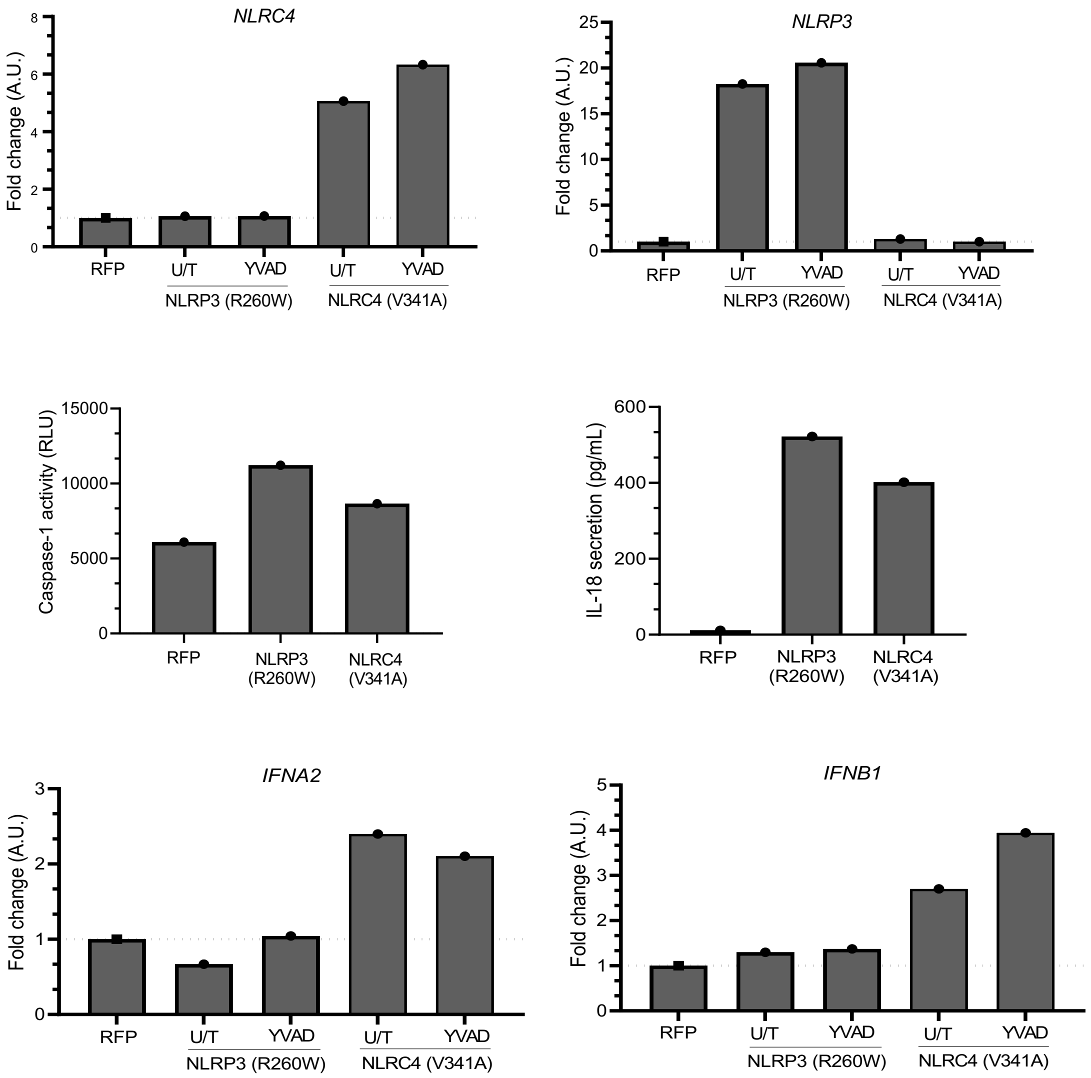

Supplemental Figure 10: NLRC4 (V341A) GOF mRNA transfections in human primary monocytes. mRNA transfections of NLRC4 (V341A), or NLRP3 (R260W), or control RFP, in human primary monocytes, cultured in the presence or absence of the Caspase-1 inhibitor YVAD as indicated. Fold changes of gene expression levels of *NLRC4* and *NLRP3* as control (top), or *IFNA2* and *IFNB1* Type-I IFN genes induced by each mRNA transfection as indicated (bottom). As control of NLR-mediated inflammasome activation, Caspase-1 activity and IL-18 secretion induced by each mRNA transfection as indicated (middle). Data representative of two different donors with similar pattern.

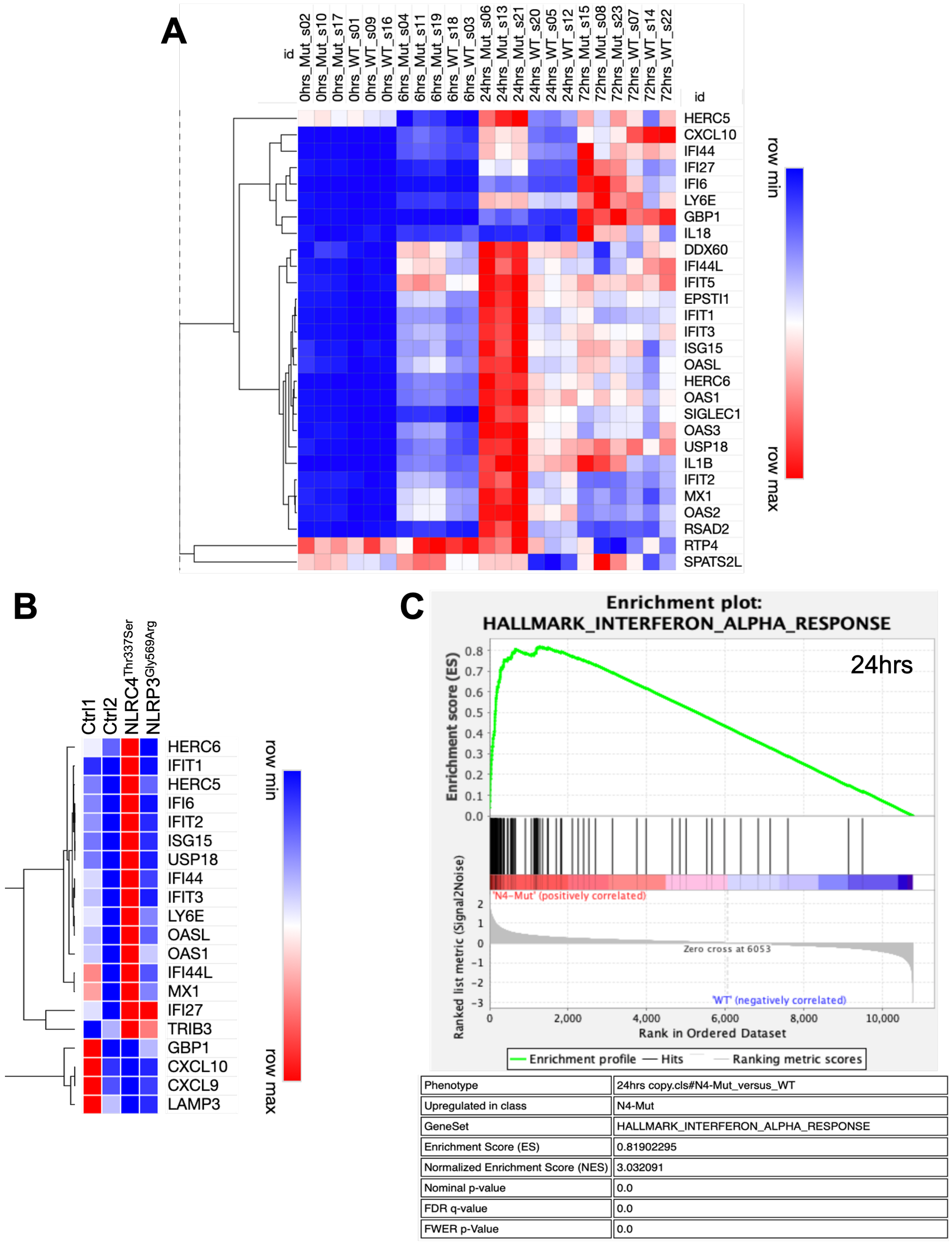

Phenotype	24hrs copy.cls#N4-Mut_versus_WT
Upregulated in class	N4-Mut
GeneSet	HALLMARK_INTERFERON_ALPHA_RESPONSE
Enrichment Score (ES)	0.81902295
Normalized Enrichment Score (NES)	3.032091
Nominal p-value	0.0
FDR q-value	0.0
FWER p-Value	0.0

Supplemental Figure 11: Type-I IFN signaling genes induced by NLRC4 (T337S) GOF mutation. (A) Expression (TPM) of 28 Type-I IFN-stimulated genes in THP1 cells stably-transduced with WT or T337S mutant NLRC4 and stimulated with PMA for the indicated timepoints. (B) Heatmap of TPM values from unstimulated primary monocyte-derived macrophages. Genes were selected from those with FC>2 of either patient versus average of controls that were also present in Type-I or Type-II IFN-stimulated genes. (C) Gene Set Enrichment Analysis of same data as in (A) for hallmark IFN alpha response genes.

CXCL10

A

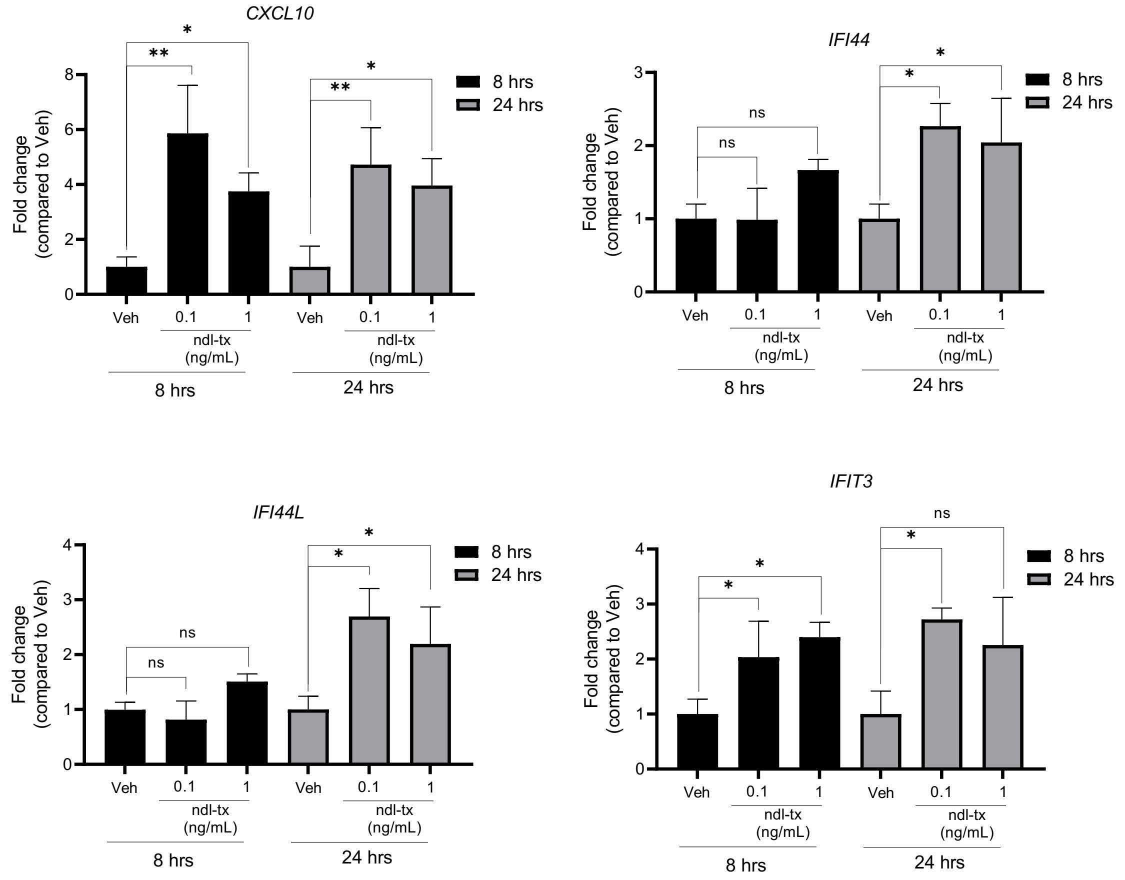
CXCL10
IFI44
IFI44L
IFIT3
Fold change (compared to Veh)
8 hrs
24 hrs
Veh
0.1
1
ndl-tx (ng/mL)
ns
*
**

B

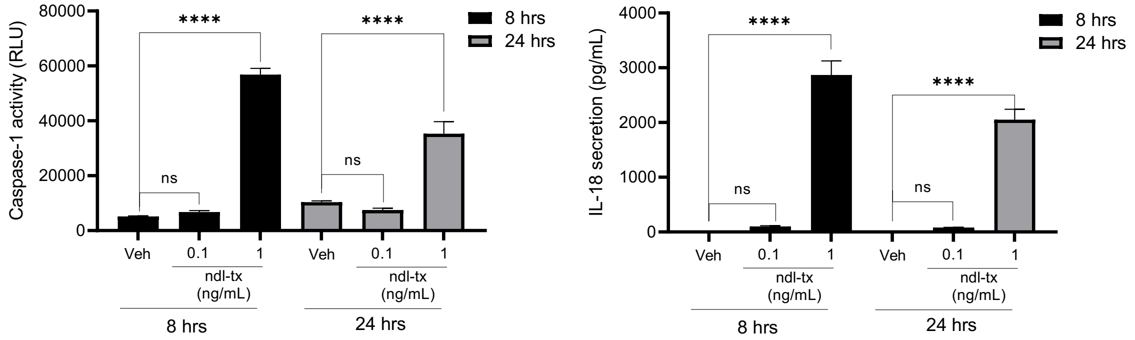
Caspase-1 activity (RLU)
IL-18 secretion (pg/mL)
8 hrs
24 hrs
Veh
0.1
1
ndl-tx (ng/mL)
ns
****

Supplemental Figure 12: Stimulation of human primary monocytes with NLRC4 trigger needle-tox leads to induction of ISGs. Human primary Monocytes were isolated from peripheral blood and rested overnight prior to stimulation with vehicle or the indicated concentrations of needle-tox (LF-N-ndl/PA) for 8 or 24 hrs. (A) Relative mRNA levels of Type-I IFN genes *CXCL10*, *IFI44*, *IFI44L* and *IFIT3* were determined by RT-qPCR. Data are plotted as fold change versus respective vehicle control for each timepoint +/- SD (n=3; 2 independent experiments). (B) Caspase-1 activity and IL-18 secretion were measured in the supernatants of stimulated cells. ns=not significant, *P<0.05, **P<0.01, ****P<0.0001.

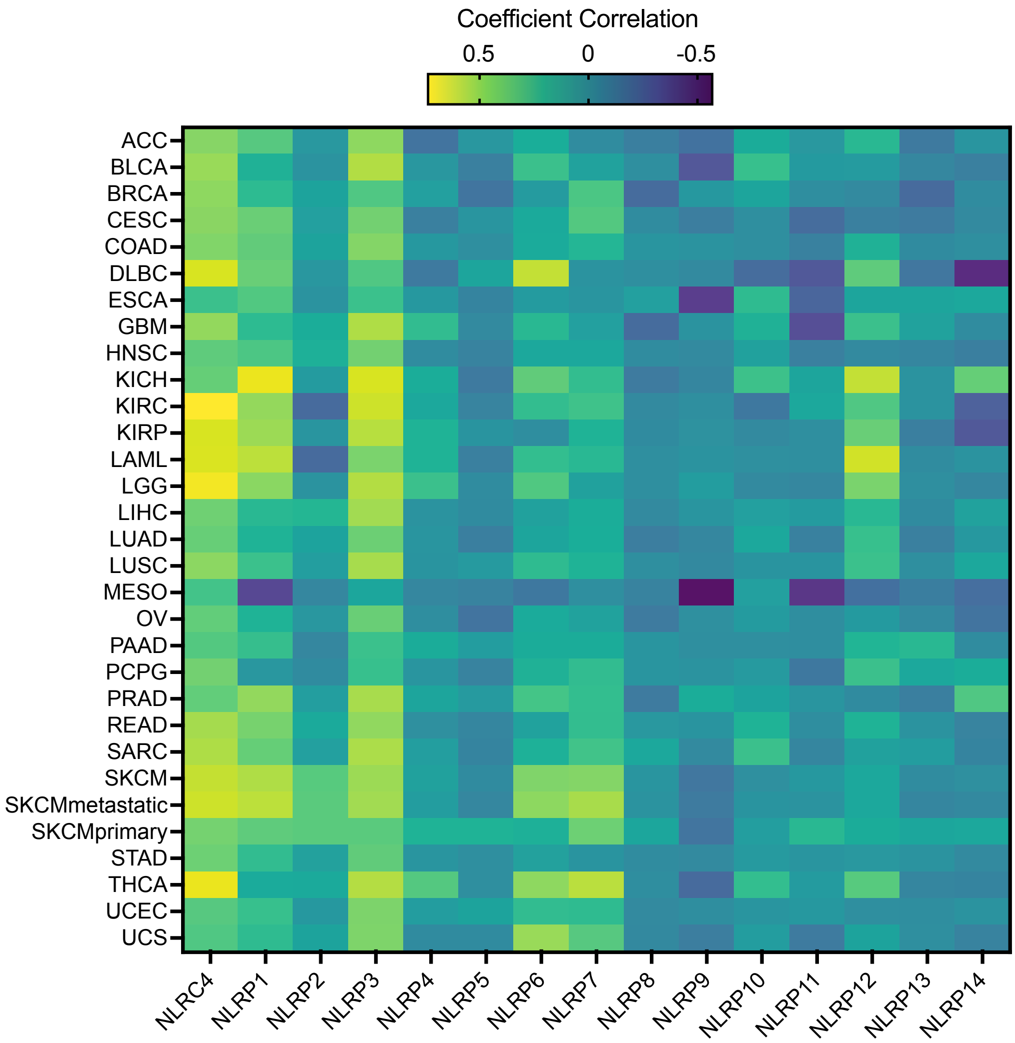

Supplemental Figure 13

Corr. with NLRC4 (pvalues)

Source	DC subset	COAD	READ	LUSC	LUAD
TCIA	pDC	0.24(2.1e-7)	0.33(1.2e-5)	0.32(1.3e-13)	0.5(0)
	iDC	0.39(0)	0.12(0.11)	0.21(1.7e-6)	0.33(4.9e-15)
	aDC	0.43(0)	0.37(7.6e-7)	0.67(0)	0.55(0)
CIBERSORT	DC resting	0.74(0)	0.72(0)	0.8(0)	0.78(0)
	DC activated	0.7(0)	0.7(0)	0.75(0)	0.68(0)
TIMER	DC	0.64(5.7e-49)	0.59(1.2e-14)	0.74(3.9e-82)	0.72(3.0e-80)

Corr. with TMEM173 (pvalues)

Source	DC subset	COAD	READ	LUSC	LUAD
TCIA	pDC	0.05(0.29)	0.06(0.44)	0.31(2.8e-12)	0.39(0)
	iDC	0.07(0.13)	0.1(0.21)	0.19(2.5e-5)	0.17(7.6e-5)
	aDC	0.37(0)	0.35(4.5e-6)	0.48(0)	0.38(0)
CIBERSORT	DC resting	0.23(6.9e-7)	0.17(0.03)	0.41(0)	0.41(0)
	DC activated	0.28(1.2e-9)	0.16(0.04)	0.49(0)	0.29(6.0e-12)
TIMER	DC	0.26(1.2e-7)	0.18(3.2e-2)	0.39(1.4e-18)	0.32(7.6e-13)

Supplemental Table 2

Supplemental Figure 13: Expression correlation between NLR family members and Type-I IFN gene signature in cancer patients. Correlation analysis was performed between the expression of each NLR gene and Type-I IFN gene signature in each tumor type in the TCGA patient dataset. Correlation coefficient R is shown in the heatmap.

Supplemental Table 2: Expression correlation between *NLRC4* or *TMEM173* (STING) and various tumor infiltrating DC subsets in colorectal (COAD, READ), or cancer patients (LUSC, LUAD), or melanoma SKCM. The various DC subset gene signatures used were obtained from various datasets including TCIA, CIBERSORT, and TIMER. Table show coefficient correlations and pvalues.

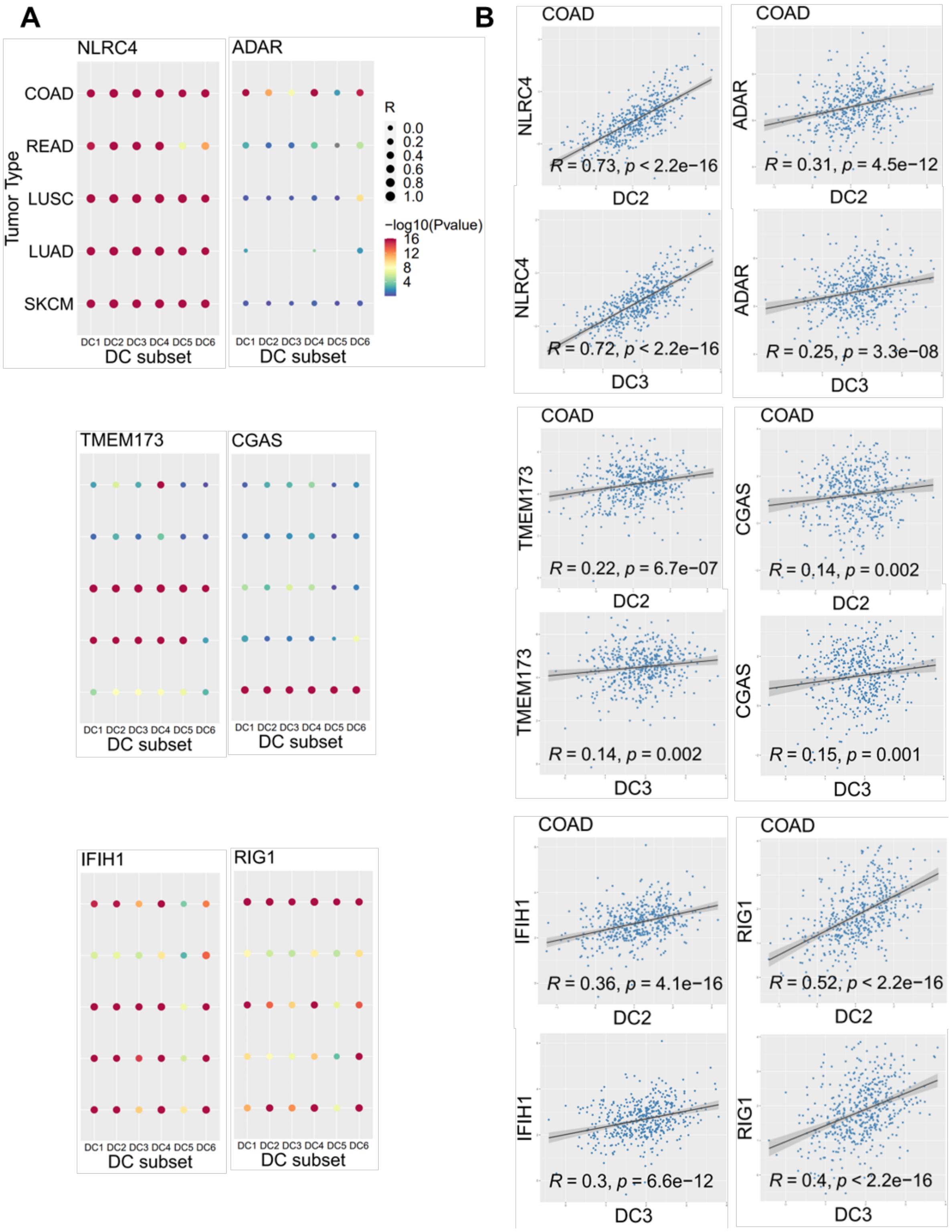
A
NLRC4
ADAR
COAD
READ
LUSC
LUAD
SKCM
Tumor Type
R
0.0
0.2
0.4
0.6
0.8
1.0
−log10(Pvalue)
16
12
8
4
DC1 DC2 DC3 DC4 DC5 DC6
DC subset
TMEM173
CGAS
IFIH1
RIG1
B
COAD
R = 0.73, p < 2.2e−16
R = 0.31, p = 4.5e−12
R = 0.72, p < 2.2e−16
R = 0.25, p = 3.3e−08
R = 0.22, p = 6.7e−07
R = 0.14, p = 0.002
R = 0.14, p = 0.002
R = 0.15, p = 0.001
R = 0.36, p = 4.1e−16
R = 0.52, p < 2.2e−16
R = 0.3, p = 6.6e−12
R = 0.4, p < 2.2e−16
DC2
DC3

Supplemental Figure 14: *NLRC4* expression is associated to tumor infiltrating DC subsets in cancer patients. (A) Expression correlation between IFN inducer genes and various tumor infiltrating DC subsets in colorectal cancer patients (COAD, READ), or lung (LUSC, LUAD), or melanoma SKCM. The various DC subset gene signatures used (DC1, DC2, DC3, DC4, DC5, DC6) were obtained from scRNAseq of human blood. (B) scatter plots showing correlation of gene expression between IFN inducer genes and DC2 or DC3 gene signatures in COAD patient tumors. COAD datasets used were obtained from the TCGA cohort.

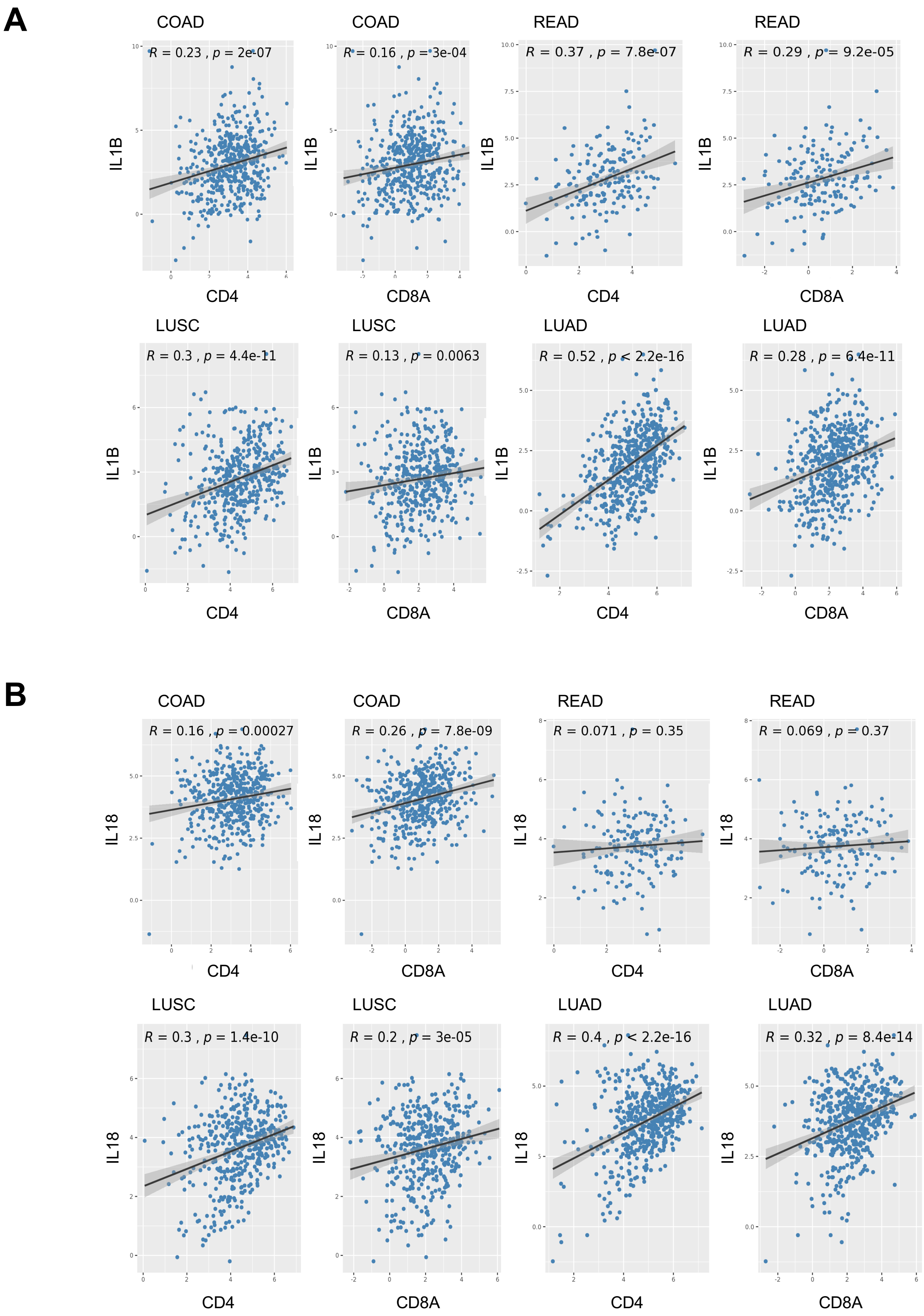
A
COAD
R = 0.23 , p = 2e-07
IL1B
CD4
COAD
R = 0.16 , p = 3e-04
IL1B
CD8A
READ
R = 0.37 , p = 7.8e-07
IL1B
CD4
READ
R = 0.29 , p = 9.2e-05
IL1B
CD8A
LUSC
R = 0.3 , p = 4.4e-11
IL1B
CD4
LUSC
R = 0.13 , p = 0.0063
IL1B
CD8A
LUAD
R = 0.52 , p < 2.2e-16
IL1B
CD4
LUAD
R = 0.28 , p = 6.4e-11
IL1B
CD8A
B
COAD
R = 0.16 , p = 0.00027
IL18
CD4
COAD
R = 0.26 , p = 7.8e-09
IL18
CD8A
READ
R = 0.071 , p = 0.35
IL18
CD4
READ
R = 0.069 , p = 0.37
IL18
CD8A
LUSC
R = 0.3 , p = 1.4e-10
IL18
CD4
LUSC
R = 0.2 , p = 3e-05
IL18
CD8A
LUAD
R = 0.4 , p < 2.2e-16
IL18
CD4
LUAD
R = 0.32 , p = 8.4e-14
IL18
CD8A

Supplemental Figure 15: Correlation between gene expression of *IL1B*/*IL18* cytokine genes and *CD4* or *CD8A* in cancer patients. Scatter plots showing correlation of gene expression between (A) *IL1B* or (B) *IL18* and *CD4* or *CD8A* in colorectal (COAD, READ) and lung cancer patients (LUSC, LUAD). Patient datasets used from the TCGA cohort. Correlation coefficients R and pvalues are indicated. COAD, colon adenocarcinoma; READ, Rectum Adenocarcinoma; LUAD, lung adenocarcinoma; LUSC, lung squamous cell carcinoma.

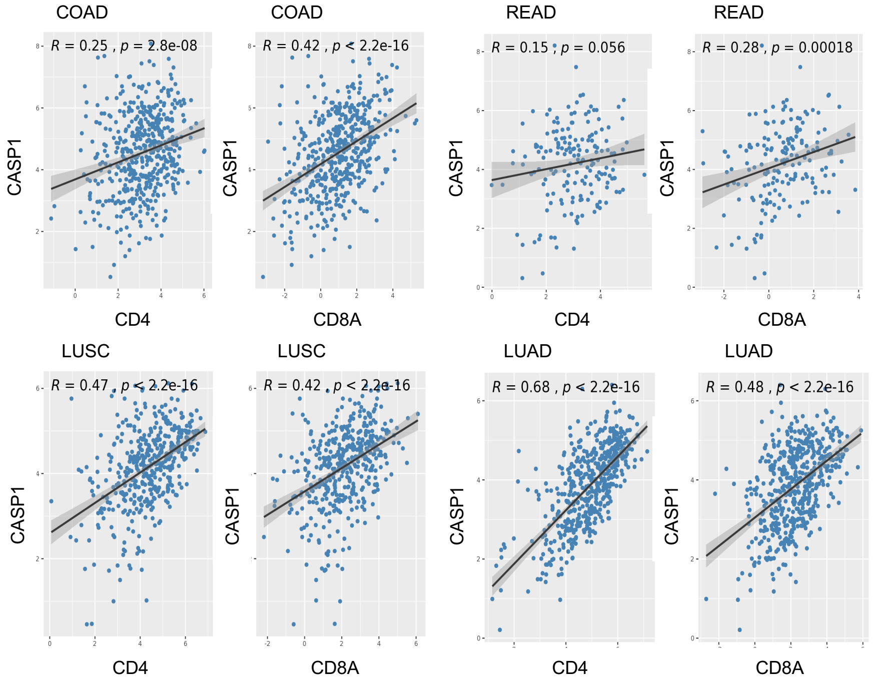

Supplemental Figure 16

	COAD		READ		LUSC		LUAD		
	Corr	pvalue	Corr	pvalue	Corr	pvalue	Corr	pvalue	
CASP1	0.25	2.80E-08	0.15	0.056	0.47	<2.2e-16	0.68	p<2.2e-16	*CD4*
	0.42	<2.2e-16	0.28	0.00018	0.42	<2.2e-16	0.48	p<2.2e-16	*CD8A*
IL1B	0.23	2.00E-07	0.37	7.80E-07	0.3	4.40E-11	0.52	p<2.2e-16	*CD4*
	0.16	3.00E-04	0.29	9.20E-05	0.13	0.0063	0.28	6.40E-11	*CD8A*
IL18	0.16	0.00027	0.071	0.35	0.3	1.40E-10	0.4	p<2.2e-16	*CD4*
	0.26	7.80E-09	0.069	0.37	0.2	3.00E-05	0.32	8.40E-14	*CD8A*

Supplemental Table 3

Supplemental Figure 16: Correlation between gene expression of *CASP1* and *CD4* or *CD8A* in cancer patients. Top, scatter plots showing correlation of gene expression between (A) *CASP1* and *CD4* or *CD8A* in colorectal (COAD, READ) and lung cancer patients (LUSC, LUAD). Patient datasets used from the TCGA cohort. Correlation coefficients R and pvalues are indicated.

Supplemental Table 3: Table summarizing results of expression correlation (coefficient R; pvalues) from scatter plots shown for *CASP1*, *ILB* and *IL18* with *CD4* or *CD8A*. COAD, colon adenocarcinoma; READ, Rectum Adenocarcinoma; LUAD, lung adenocarcinoma; LUSC, lung squamous cell carcinoma.

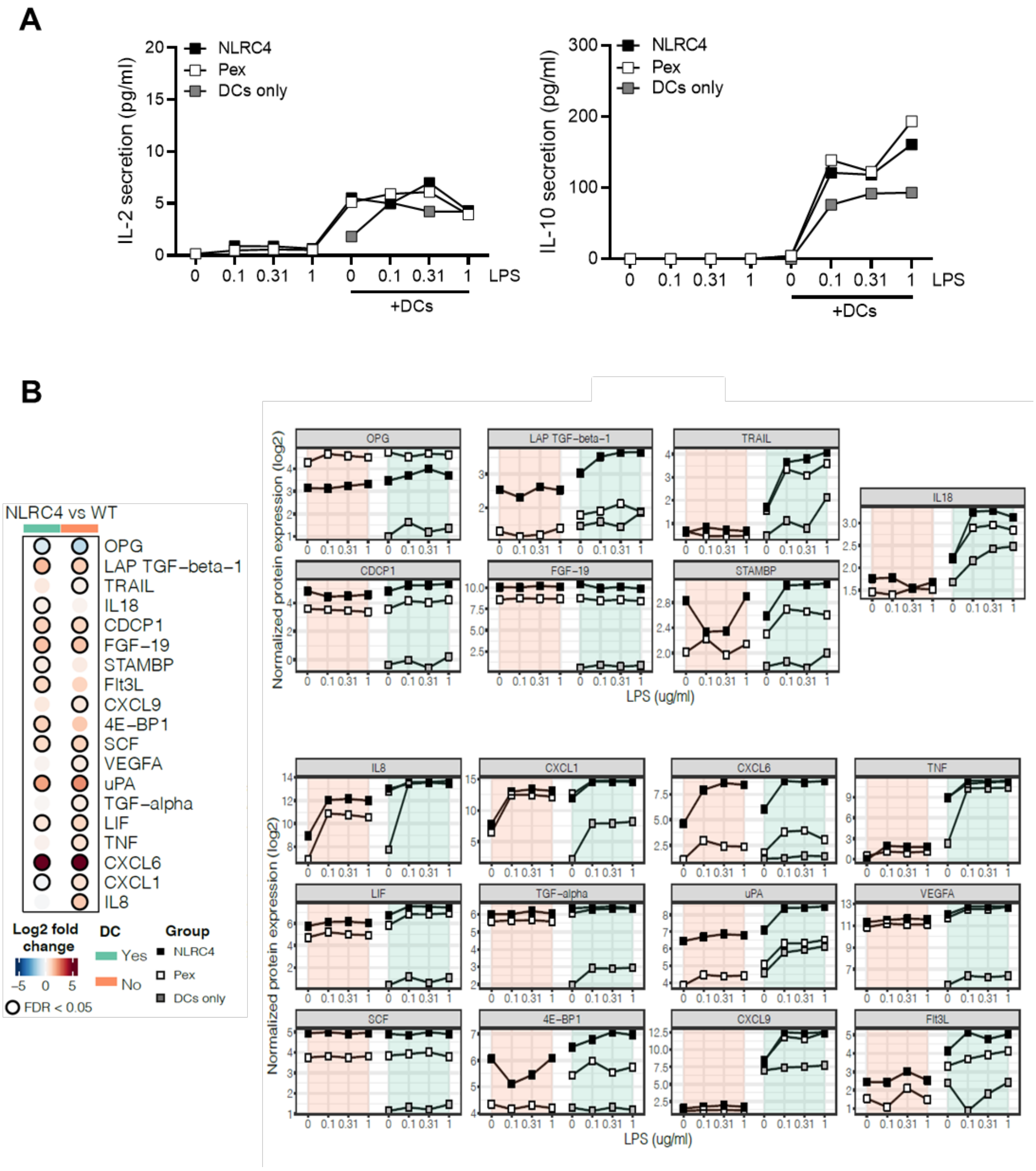
A
IL-2 secretion (pg/ml)
IL-10 secretion (pg/ml)
NLRC4
Pex
DCs only
LPS
+DCs
B
NLRC4 vs WT
OPG
LAP TGF−beta−1
TRAIL
IL18
CDCP1
FGF−19
STAMBP
Flt3L
CXCL9
4E−BP1
SCF
VEGFA
uPA
TGF−alpha
LIF
TNF
CXCL6
CXCL1
IL8
Log2 fold change
DC
Yes
No
Group
NLRC4
Pex
DCs only
FDR < 0.05
Normalized protein expression (log2)
LPS (ug/ml)

Supplemental Figure 17: Secretomics analysis of NLRC4-mediated DC maturation (A) IL-2 and IL-10 secretion from cell culture supernatants measured by MSD from cultures of HT29-NLRC4 cell line, or HT29-pEx control cell line, or co-cultured with primary DCs isolated from human blood, with or without LPS (µg/ml). Co-cultures of HT29/DCs (1/1.2 ratio) were maintained for 24hrs, in the presence or not of LPS. Data representative of 2 donors with similar pattern. (B) Same experiment as in (A) but additional differentially secreted proteins as measured by Olink proteomics. Plots show the normalized protein expression values of the various markers (log2); co-cultures with DCs (green) or not (orange). Heatmap to the left indicates the difference between the fold changes in cells expressing NLRC4 or the empty vector, and statistically significant changes after multiple testing are circled in black.

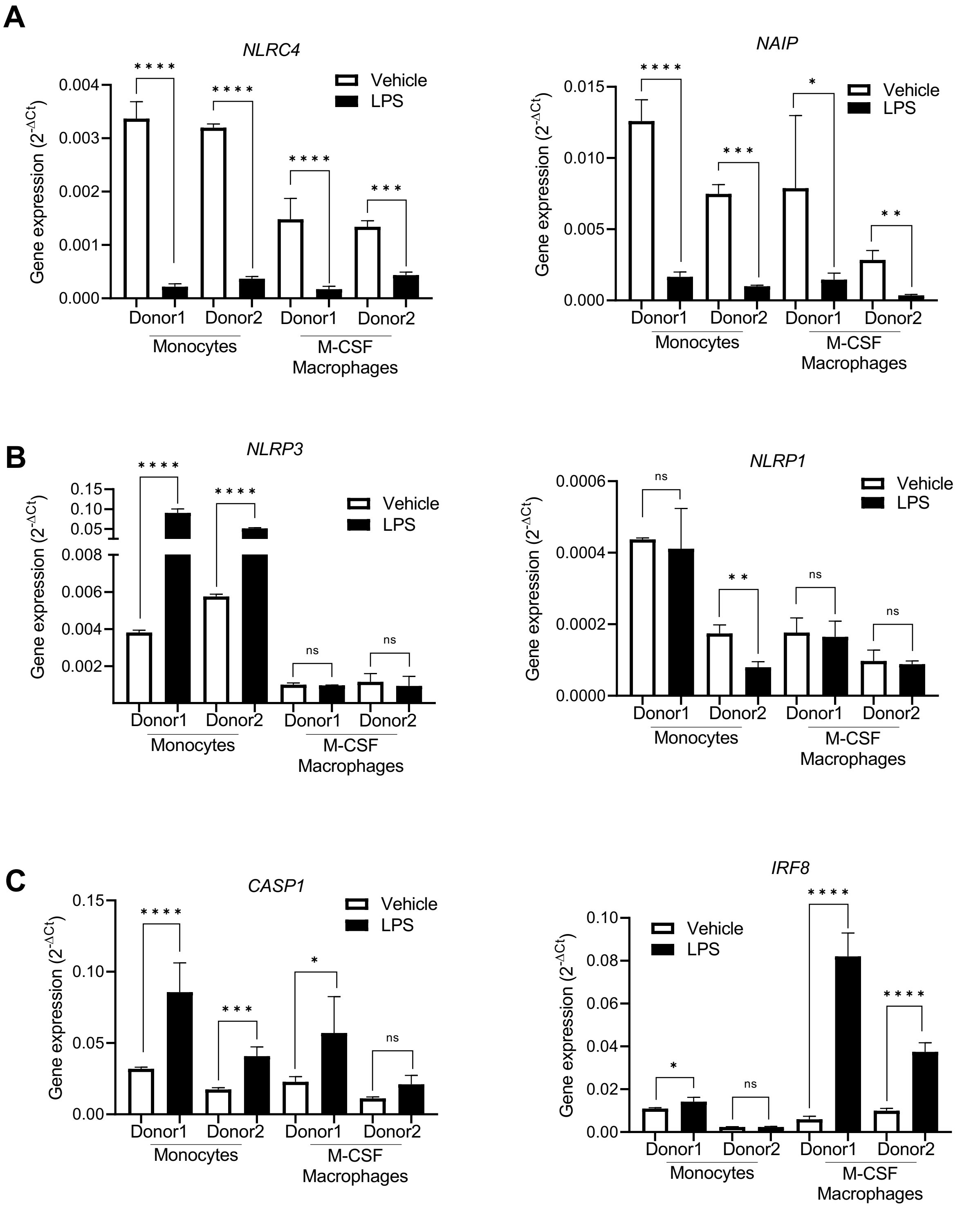

Supplemental Figure 18: LPS specifically impairs gene expression of *NLRC4* and *NAIP*, but not other NLR family members. Human primary monocytes or M-CSF-differentiated macrophages were treated or not with LPS and gene expression of *NLRC4* and *NAIP* (A), or *NLRP3* and *NLRP1* (B), or *CASP1* and *IRF8* (C) was determined by Q-PCR. Data are normalized to a housekeeping gene and expressed as the mean +/- SD (n=3; 2 independent experiments); *P<0.05, **P<0.01, ***P<0.001, ****P<0.0001.
