# Supplementary figures and images for: Human NLRC4 expression promotes cancer survival and associates with type I interferon signaling and immune infiltration
Source: J Clin Invest. 2024 Apr 23;134(11):e166085. doi: 10.1172/JCI166085 (PMC11142746; doi:10.1172/JCI166085)

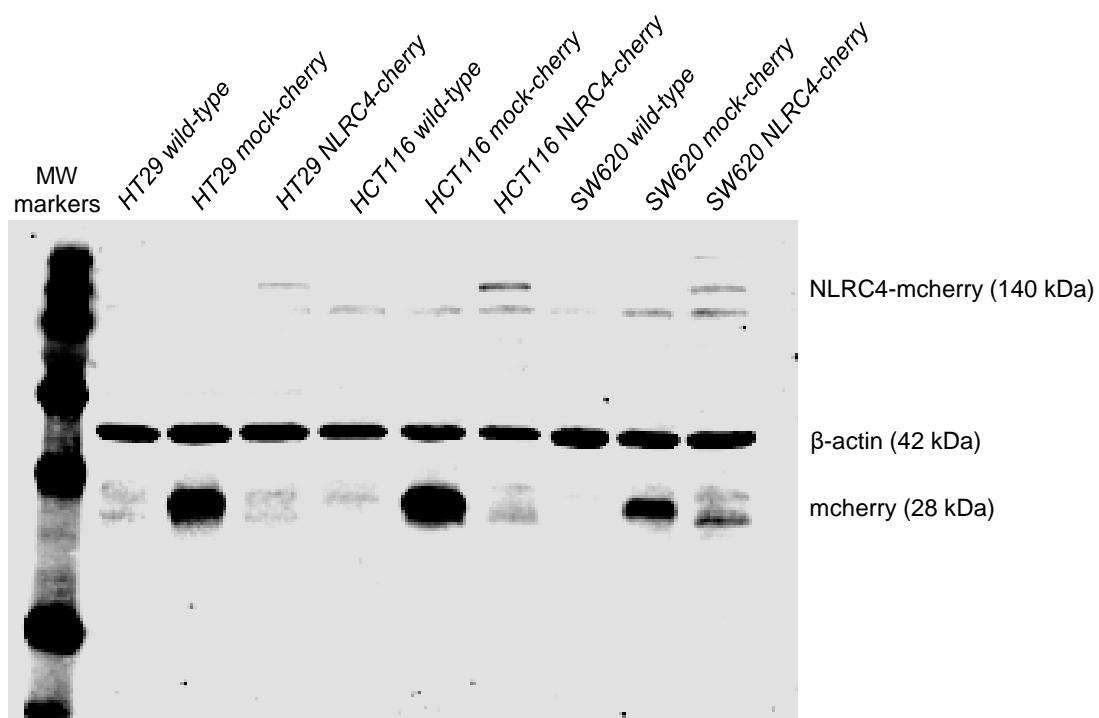

Unedited Blot  
Supplemental Figure 9

Supplement: Unedited blot and gel images [file jci-134-166085-s233.pdf]
